# What drives the cost of biowaste-to-lipase production? Linking titre, rate and yield to techno-economic performance in batch-to-continuous *Pichia pastoris* fermentation

**DOI:** 10.1186/s40643-026-01153-x

**Published:** 2026-10-29

**Authors:** Ana G. Del Hierro, Violine Raynal, Ruth Ordóñez, Fuensanta Verdú-Navarro, Eoin Casey

**Affiliations:** 1 School of Chemical and Bioprocess Engineering, University College Dublin, Belfield, Dublin 4, Ireland; 2 BiOrbic, Research Ireland Centre for Bioeconomy, Dublin, Ireland; 3 Bioprocessing R&D Department, Parque Tecnológico Fuente Álamo, Bionet, Fuente Álamo, Spain

**Keywords:** *Candida rugosa lipase* type 1, *Pichia pastoris*, Recombinant enzyme production, Techno-economic assessment, Fruit and vegetable waste, Biowaste-derived carbon sources, Continuous fermentation, SuperPro designer, Circular bioeconomy

## Abstract

Fruit and vegetable (FV) waste represents an underutilized organic stream in Europe, yet its soluble sugar fraction remains poorly exploited as a bioprocess feedstock. Recombinant enzyme production from biowaste is often bottlenecked by heterogeneous substrate composition, low fermentable sugar titres, and uncertain process economics. This study evaluates the techno-economic feasibility of recombinant *Candida rugosa* lipase type 1 (CRL1) production in *Pichia pastoris* (*Komagataella phaffii*) under the constitutive glyceraldehyde-3-phosphate dehydrogenase promoter *pGAP* across three carbon sources: commercial glucose (Scenario A), mechanically pressed fruit and vegetable juice (Scenario B), and an enzymatically hydrolysed, nanofiltered fruit and vegetablesugar stream (Scenario C). Bench-scale fermentation kinetics were integrated into 24 SuperPro Designer models across four scales (1 to 5000 L nominal vessel capacity; 0.75 to 3750 L working volume) under batch and continuous chemostat ($$\:D=0.05 {\mathrm{h}}^{-1}$$) modes. Interpreted through the Titre, Rate, and Yield (TRY) framework, continuous operation on glucose increased volumetric CRL1 productivity 12.6-fold over batch (0.084 vs. 0.06 U L^−1^ h^−1^. Conversely, continuous operation on the hydrolysed stream did not improve productivity over batch, indicating potential kinetic inhibition from enzymatic pretreatment products or suboptimal dilution rate. At the 5000 L vessel scale under continuous mode, unit production costs were $1186, $36,049, and $3416 $$\:{\mathrm{kg}}^{-1}$$ for Scenarios A, B, and C, respectively, all exceeding the commodity lipase benchmark ($25 $$\:{\mathrm{kg}}^{-1}$$). Facility-dependent costs dominated Scenarios A and B (73% and 78% of OPEX), whereas enzyme reagents dominated Scenario C (75%). Sensitivity analysis confirmed that secreted protein titre and downstream recovery efficiency are the primary economic drivers, demonstrating that feedstock acquisition cost is not the binding constraint. Early-stage TEA driven by bench-scale kinetic parameterization provides essential screening prior to pilot-scale investment in biowaste-to-enzyme biorefineries.

**Graphical abstract:**

**Figure:**
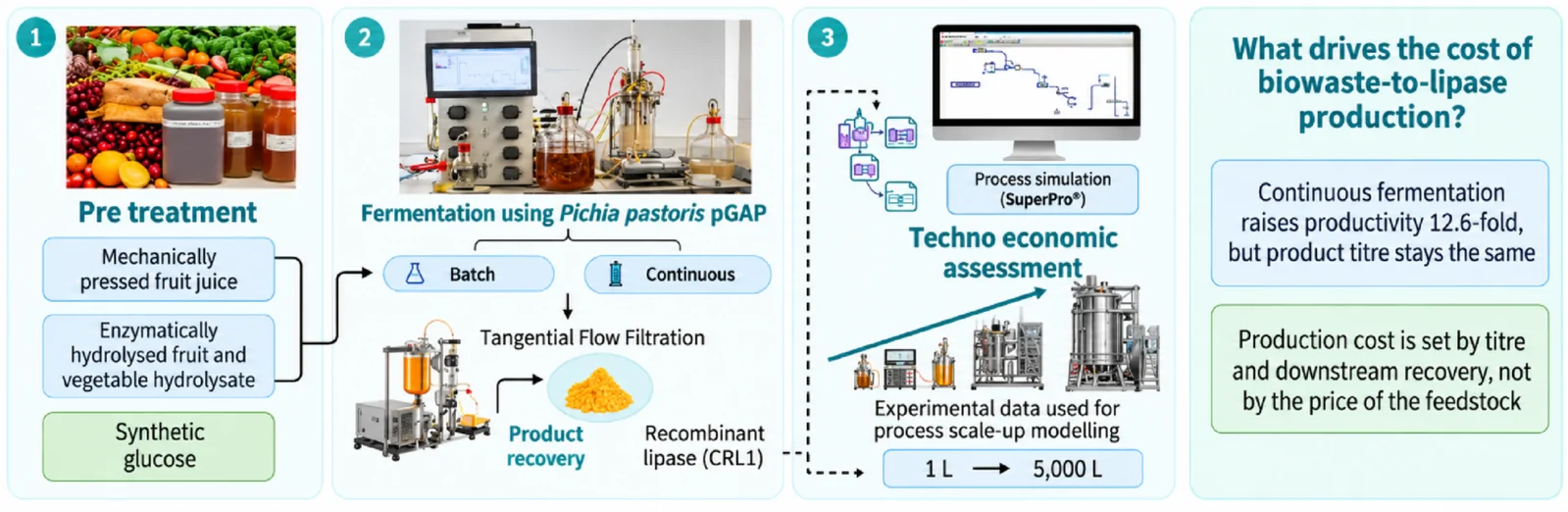

**Supplementary Information:**

The online version contains supplementary material available at https://doi.org/10.1186/s40643-026-01153-x.

## Introduction

The transition to a circular bioeconomy requires the valorisation of high-volume organic side streams into functional, high-value bioproducts (Liakou et al. 2018; Sagar et al. 2018; Ganesh et al. 2022). Fruit and vegetable (FV) waste represents an attractive carbon platform due to its high concentration of soluble carbohydrates (monomeric fructose, glucose, and sucrose). However, the use of unrefined streams as fermentation substrates is limited by low initial sugar titres and complex matrix impurities (Ganesh et al. 2022).

Whether FV waste streams are economically viable fermentation feedstocks depends not on sugar content but on the full cost chain: pretreatment, solids removal, water consumption, downstream recovery and scale-dependent capital (Cristóbal et al. 2018; Muhammad and Rosentrater 2020; Sabater et al. 2020; Elgarahy et al. 2023). Under EU Directive 2018/851, biowaste is defined by its origin, rather than by the intensity of subsequent processing. (European Parliament 2018).

Recombinant enzyme production is a demanding test case for this comparison because unit production cost must be compatible with industrial enzyme markets where margins are narrow. Lipases are high-demand industrial hydrolases used in detergents, food processing, oleochemistry and pharmaceuticals (Chandra et al. 2020; Ali et al. 2023). *Candida rugosa* lipase type 1 (CRL1; EC 3.1.1.3) is the dominant isoform in commercial preparations, with broad substrate specificity, interfacial activation and alkaline stability relevant to detergent applications (Chandra et al. 2020; Abdelaziz et al. 2025). Previous techno-economic assessments of recombinant enzyme production have consistently found that production costs are driven primarily by upstream titre, fermentation scale, and downstream recovery efficiency, while feedstock price has a comparatively minor impact. This trend has been demonstrated for β-glucosidase production in *E. coli* (Ferreira et al. 2018), cellulase in *Trichoderma reesei* (Taiwo et al. 2022) and formate dehydrogenase in bacterial hosts (Cunniffe et al. 2025). For waste-derived substrates, Muhammad and Rosentrater (2020) showed that low feedstock cost is routinely offset by equipment, membrane area, water treatment and facility-dependent overhead.

In this context, the titre, rate and yield (TRY) framework provides a useful basis for linking fermentation performance with process economics. Titre determines product concentration and downstream processing burden; rate determines reactor utilisation and annual production capacity; and yield determines raw-material demand (Konzock and Nielsen 2024). For waste-derived enzyme production, this distinction is particularly important because a low-cost carbon source does not necessarily translate into economic viability if product titre, volumetric productivity or downstream recovery remain limiting. Therefore, interpreting techno-economic outputs through TRY helps distinguish whether process economics are constrained by biological expression, operating mode, substrate conversion or downstream processing. While the TRY framework has been proposed conceptually, its empirical application to a real waste-derived bioprocess spanning multiple operating modes and scales remains limited; this study applies it directly to experimental and simulated data across batch and continuous operation. To the best of our knowledge, this is one of the first studies to apply the TRY framework empirically to real waste-derived fermentation data spanning both batch and continuous operating modes across industrial scales, rather than to defined synthetic media or purely conceptual cost models.

*Pichia pastoris (formally reclassified as Komagataella phaffii)* (Kurtzman 2009) under the constitutive *glyceraldehyde-3-phosphate dehydrogenase* (*pGAP*) promoter is a methanol-free expression system for *CRL1* (García-Ortega et al. 2019) in which specific productivity is growth-rate dependent (Rebnegger et al. 2014). Fed-batch, the conventional intensification strategy for this yeast, requires concentrated feed solutions of approximately 500 g L⁻¹ glucose (Stratton et al. 1998). However, the waste-derived streams used for carbon replacement in this study reached a physical upper limit of only 78 g L⁻¹ total sugars after preprocessing. Concentrating this biowaste stream up to the 500 g L⁻¹ industrial threshold is technically and energetically unfeasible, effectively ruling out conventional fed-batch operation as a viable option for this feedstock. Consequently, this study evaluates and compares batch versus continuous configurations to identify an efficient processing alternative. Continuous chemostat operation was studied as the intensification strategy, as it theoretically maximizes volumetric productivity and maintains steady-state performance under nutrient limitation without requiring highly concentrated feed inputs (Xie 2022). To date, studies on waste-derived substrates for recombinant protein production have focused predominantly on fermentation performance (Sagar et al. 2018; Do et al. 2019), with limited integration of pretreatment economics, continuous operation, and industrial-scale cost modelling into a single comparative model. Although separate economic models exist for generic biorefineries (Eggeman and Elander 2005) and conventional enzyme production (Muhammad and Rosentrater 2020), their integration with empirical continuous data from raw biowaste remains unaddressed. This study tackles this knowledge gap by determining how FV waste-derived carbon-source processing affects the techno-economic feasibility of recombinant CRL1 production in *P. pastoris*, using waste-derived substrates sourced within the Horizon Europe Mix Matters project as case studies. Three carbon sources, commercial glucose (Scenario A), mechanically pressed fruit and vegetable (FV) juice (Scenario B) and an enzymatically hydrolysed and nanofiltered FV sugar stream (Scenario C), were evaluated for recombinant CRL1 production *in Pichia pastoris* under constitutive *pGAP* promoter across batch, and at bench scale (0.75–3 L). Bench-scale fermentation data from batch and chemostat (D = 0.05 h⁻¹) operation were integrated into 24 SuperPro Designer process models spanning four fermenter scales (ranging from 1 to 5000 L). The analysis focuses on defining the biological expression limits and downstream unit operation boundaries required to assess the requirements for commercial implementation of biowaste-to-lipase processes.

## Materials and methods

### Process scope and scenarios

Three carbon-source scenarios were evaluated for recombinant CRL1 production in *P. pastoris pGAP* commercial glucose as a defined reference (Scenario A), mechanically pressed FV juice as a biowaste-derived substrate (Scenario B), and an enzymatically hydrolysed and nanofiltered FV sugar stream as a processed carbon source (Scenario C). The mechanically pressed FV juice (Scenario B) was sourced from mixed fruit and vegetable biowaste (pear, apple, strawberry, banana, tomato, pepper, lettuce, cucumber, cauliflower, courgette) and pretreated by ILVO (Belgium) using a spiral-filter press, as part of the MixMatters project (April 2025). This pilot batch was selected as representative of the compositional profile of mixed FV biowaste generated at wholesale markets, such as Mercavalencia (Valencia, Spain), the intended source stream for this application within the project. The enzymatically hydrolysed NF FV sugar stream (Scenario C) was sourced from mixed fruit and vegetable biowaste (peach, papaya, apple, pear, kiwi, tangerine, plum, banana, lemon, aubergine, tomato, onion, cucumber, lettuce, artichoke, pepper, carrot) collected via Mercavalencia (Valencia, Spain) and pretreated by AINIA (Spain) through shredding, grinding, leaching, enzymatic hydrolysis with cellulase and hemicellulase, and nanofiltration (November 2024). The specific commercial enzyme formulation used for hydrolysis is proprietary information held by AINIA and could not be disclosed for this publication.

Under Article 3(4) of Directive 2008/98/EC, as amended by Directive (EU) 2018/851, biowaste is defined by its origin, including biodegradable waste from wholesale premises, rather than by the intensity of subsequent processing (European Parliament 2018). Both waste-derived feedstocks are representative of mixed FV biowaste generated at wholesale markets such as Mercavalencia and are therefore modelled at zero raw-material acquisition cost regardless of the pretreatment route applied. The additional processing intensity of Scenario C is reflected in the capital and operating expenditure of the corresponding unit operations within the process flowsheet, not in a raw-material price.For each scenario, batch and chemostat (D = 0.05 h⁻¹) operation were compared to evaluate the effect of fermentation mode on CRL1 production and process economics. The experimental and modelling structure is summarised in Table 1.

All scenarios shared the same general upstream and downstream process structure but differed in the carbon-source preparation stage. Where experimental continuous data were not available, continuous operation was included as a model-based projection and interpreted separately from experimentally validated cases.

**Table 1:** Experimental and modelling structure used to distinguish the effect of fermentation mode from the effect of feedstock pretreatment intensity

Scenario	Feedstock	Experimental mode	Continuous mode
A	Commercial glucose	Batch vs. chemostat	Experimentally validated Chemostat
B	Mechanically pressed FV juice	Batch only	Model-based projection
C	Enzymatically hydrolysed NF sugar stream	Batch vs. chemostat	Experimentally validated Chemostat

### Experimental baseline 

#### Inoculum and media preparation

Unless otherwise stated, all reagents used in this work were purchased from Duchefa Biochemie (Haarlem, The Netherlands). A recombinant *P. pastoris* strain expressing CRL1 under the control of the constitutive *pGAP* promoter was used as production host. (10 g L⁻¹ yeast extract, 20 g L⁻¹ peptone, 20 g L⁻¹ dextrose) at 4 °C, and working cell banks were prepared in liquid YPD supplemented with 15% (v/v) glycerol and stored at − 20 °C until use, following standard procedures for recombinant *P. pastoris* cultivation (Waterham et al. 1997; Stratton et al. 1998). The bioreactor was inoculated at 5% (v/v) using a 24-hour shake-flask culture prepared in baffled flasks with YPD medium ( at 28 ± 1 °C, 200 rpm), standardising the inoculum age at an OD_600_ of approximately 30.

For bioreactor experiments and process simulations, two complementary media types were defined: sugar-rich substrate media—differentiated into batch substrate medium (SUBS-batch) and continuous feed medium (SUBS-feed)—and a salt solution (PMS-I), adapted from previously described formulations for *P. pastoris* (García-Ortega et al. 2019). The composition of the raw waste-derived feedstocks used to formulate both SUBS-batch and SUBS-feed in Scenarios B and C is detailed in Table 2.

**Table 2:** Composition of waste-derived sugar feedstocks used in Scenarios B and C. PAN: primary amino nitrogen. NF, nanofiltration concentrate

Feedstock	Total sugars (g/L)	Glucose (g/L)	Fructose (g/L)	Other sugars	NH4+ (g/L)	PAN (g/L)	Solids (%)
Mecanically pressed Fruit and vegetable juice	78.05	25.33	51.70	1.02	0.023	0.189	2%
Enzymatically hydrolysed and nanofiltered FV sugar stream	68.04	30.51	35.28	2.25	0.106	0.537	–

To prepare the substrate media, the raw stock solutions were diluted with deionised water to obtain the target total fermentable sugar concentrations: 40 g L⁻¹ for SUBS-batch and 50 g L⁻¹ for SUBS-feed for continuous. Specifically, for SUBS-batch (40 g L⁻¹ total sugars), the raw fruit juice (78.05 g L⁻¹) and NF stream (68.04 g L⁻¹) were diluted 1.95-fold and 1.70-fold, respectively. For SUBS-feed (50 g L⁻¹ total sugars), stock solutions were diluted 1.56-fold (Scenario B) and 1.36-fold (Scenario C). For the control scenario (Scenario A), synthetic SUBS-batch and SUBS-feed were prepared by dissolving commercial D-glucose in deionised water to equivalent working concentrations (40 g L⁻¹ and 50 g L⁻¹, respectively).

For initial batch operation, SUBS-batch (40 g L⁻¹ total sugars) was prepared directly inside the fermenter vessel at approximately two-thirds of the final working volume and autoclaved at 121 °C for 30 min. For continuous operation, fresh SUBS-feed (50 g L⁻¹ total sugars) was prepared separately in external feed reservoirs and heat-sterilised prior to continuous feeding. PMS-I supplied inorganic nutrients and nitrogen and, in batch mode, contained (per litre) 1.8 g citric acid, 12.6 g (NH₄)₂HPO₄, 0.9 g KCl and 0.5 g MgSO₄·H₂O; for continuous operation, PMS-I contained 0.9 g citric acid, 4.35 g (NH₄)₂HPO₄, 1.7 g KCl and 0.65 g MgSO₄·H₂O. Trace elements and vitamins were supplied via PTM1 trace salts and biotin, sterilised by filtration (0.22 μm) and added aseptically post autoclaving (Cereghino and Cregg 2000). Sugars and salts were always sterilised separately to prevent Maillard reactions. Media pH was adjusted to 6.2 ± 0.1 for batch start-up and 4.7 ± 0.1 for continuous feeds and controlled at pH 4.7 during fermentation to ensure compatibility with downstream clarification. Bioreactor temperature was maintained at 25 °C to support CRL1 secretion under *pGAP* control (García-Ortega et al. 2019).

#### Bench-scale fermentation

Bench-scale fermentations were conducted in 1 L vessel, F1 stirred-tank bioreactor (Bionet, Spain) controlled by ROSITA^®^ software (Bionet, Spain) and coupled to a Breath off-gas analyser (Bionet, Spain) for continuous monitoring of exhaust O₂ and CO₂. Experiments were performed at 0.75 L working volume using the three feedstocks (glucose, fruit juice, and concentrated hydrolysate) in batch and batch-to-chemostat configurations and served as the primary data set for kinetic and yield parameterisation.

All fermentations were initiated in batch mode with 40 g L⁻¹ of carbon source, supplied either as glucose or as an equivalent concentration of biowaste-derived sugars. Temperature was maintained at 25 ± 1 °C, and pH was controlled at 4.7 ± 0.1 using NH₄OH. Dissolved oxygen was maintained at 30% air saturation via a cascade loop acting exclusively on the agitation speed, while the aeration rate was kept fixed at 1.5 vvm (1.125 L min^− 1^ ) with compressed air. At the 0.75 L working volume, the dissolved-oxygen cascade adjusted the impeller speed between 300 and 1800 rpm, while aeration was maintained constant at 1.5 vvm, corresponding to 1.125 L min⁻¹.Antifoam (ACEPOL 69, Dystar, Singapore) was added as required.

Continuous cultures were initiated from a batch phase (40 g/L sugar) and transitioned to continuous feeding (50 g/L fresh medium) after 18–24 h. Three dilution rates were evaluated sequentially within a single continuous cultivation: D = 0.025, 0.040 and 0.050 h⁻¹, corresponding to residence times of 40, 25 and 20 h, respectively; D = 0.05 h⁻¹ was confirmed as the operational steady state for CRL1 production. Feeding commenced when the batch carbon source was exhausted at approximately 24 h, as indicated by a drop in off-gas CO₂ and a rise in dissolved oxygen. Fresh SUBS medium containing 50 g L⁻¹ total sugars were supplied at a constant volumetric flow rate (0.0375 L h⁻¹) corresponding to a dilution rate of 0.05 h⁻¹, and the same flow rate was withdrawn to maintain constant working volume. Continuous cultures were operated for at least three residence times, and steady state was defined as the condition in which optical density and off-gas CO₂ remained within ± 5% of their mean values for three consecutive residence times, following standard chemostat practice (Doran 2012). The resulting 0.75 L online data recorded in Rosita 1.0 software (Bionet, Spain) were used to estimate specific growth rates, biomass yields on substrate, product titres, and volumetric productivities for each feedstock. Continuous fermentation was performed on glucose and concentrated feedstock only. Fruit juice was evaluated exclusively under batch conditions.

#### Downstream processing experiments

Downstream processing was evaluated experimentally on selected batches to inform model assumptions and identify potential bottlenecks. A fruit-juice batch fermentation performed at 7 L working volume was clarified and concentrated as references for scale up. Fermentation broth was first centrifuged in 100 mL bottles at 400 mL per cycle until the total volume had been processed, yielding about 4 L of clarified supernatant. The supernatant was concentrated using a tangential-flow filtration (TFF) system (Bionet, Spain) equipped with a ceramic membrane of 15 kDa nominal cut-off, targeting a theoretical concentration factor of approximately 13 (corresponding to reducing the 4 L clarified supernatant to approximately 300 mL retentate) (Verdú-Navarro et al. 2026). Samples of feed, permeate and retentate were analysed for total protein, enzymatic activity and recombinant protein expression by SDS-PAGE.

#### Analytical methods

Offline analyses were performed throughout fermentations. Optical density at 600 nm was measured using a Zuzi 4111R spectrophotometer (Daselab, Spain), with appropriate dilution to maintain absorbance below 0.9. DCW was determined by centrifuging broth samples at 9000 rpm for 30 min in a Digicen 21 centrifuge (Orto Alresa, Madrid, Spain) equipped with an RT154 rotor, collecting the supernatant for protein and activity assays, drying the pellet at 80 °C for 24 h and weighing to constant mass (Çaloğlu and Binay 2023).

Total extracellular protein was quantified by Bradford assay (Bradford 1976) for rapid at-line screening and by bicinchoninic acid (BCA) assay (Smith et al. 1985) for definitive quantification. Both assays used bovine serum albumin (BSA) as standard. Bradford reagent (Pierce Coomassie Bradford Plus, Thermo Fisher Scientific) was read at 595 nm; BCA reagent (Pierce BCA Protein Assay Kit, Thermo Fisher Scientific) was read at 562 nm, both on a Genesys 20 spectrophotometer. Bradford assay is susceptible to suppression by fermentation matrix components (Kruger 1994). BCA assay to interference from reducing sugars including glucose and fructose (Reichelt et al. 2016).

CRL1 expression was confirmed qualitatively by Sodium Dodecyl Sulfate – Polyacrylamide Gel Electrophoresis (SDS–PAGE) (Laemmli 1970). Clarified supernatant samples were denatured with loading buffer, resolved on 12% polyacrylamide gels and stained with Coomassie Blue R-250 to confirm the presence of a band at the expected molecular weight of 70 kDa, consistent with N-glycosylation of the 60 kDa unglycosylated CRL1 backbone (García-Ortega et al. 2019).

Lipase activity was determined spectrophotometrically using p-nitrophenyl butyrate (pNPB; Fluorochem, Hadfield, UK) as chromogenic substrate, modified from Barik et al. (2022). Clarified supernatant (0.1 mL) was mixed with 0.8 mL of 0.1 M sodium phosphate buffer (pH 7.0) and 0.1 mL of 10 mM pNPB prepared in acetone. The reaction was incubated at 30 °C for 30 min and terminated by addition of 0.25 mL of 0.1 M NaOH to stabilise the p-nitrophenolate chromophore (Syedd-León et al. 2020). Absorbance was measured at 410 nm; samples were diluted 1:10 or 1:50 prior to measurement where required. One unit of lipase activity (U) was defined as the amount of enzyme catalysing 1 µmol pNPB hydrolysis per minute. Activity is expressed per litre of clarified supernatant (U L⁻¹) and volumetric productivity (U L⁻¹ h⁻¹) throughout this study (Syedd-León et al. 2020). For waste-derived feedstocks, blank corrections were applied using substrate-free controls in the corresponding matrix to account for background absorbance; residual optical interference could not be fully eliminated and is acknowledged in data interpretation. Specific growth rate (µ), biomass yield on substrate (Y_x/s_), volumetric productivity and specific productivity were calculated from DCW, substrate concentration and activity measurements for each run.

### Process modelling

Twenty-four techno-economic analyses (TEA) were performed using SuperPro Designer v14 (Intelligen Inc., Scotch Plains, NJ, USA). The software was used to build steady-state mass and energy balances, equipment sizing, capital cost estimation, and profitability analysis for three carbon-source scenarios (A, B, and C) at four production scales (1, 100, 1000, and 5000 L fermenter working volume) in both batch and continuous operating modes.

Each scenario was simulated under both batch and continuous configurations across four fermenter vessel capacities of 1, 100, 1000 and 5000 L, operated at 75% working volume. Throughout this paper, scales are referred to by vessel capacity for consistency with the simulation outputs. For each scale, the process consisted of: media preparation (blending tanks V-101 to V-106; heat sterilisation ST-101, ST-102); inoculum preparation in YPD medium with seed fermentation in SFR-101 (5% v/v of main fermenter volume); batch fermentation in FR-101 for biomass establishment; continuous fermentation in FR-102 for CRL1 production; and downstream DSP comprising disk-stack centrifugation (DS-101; 99% biomass removal), pH adjustment (V-104, pH 4.7), and crossflow microfiltration (MF-101; 0.2 μm membrane; 85% CRL1 recovery).

For Scenario C, the 5000 L batch configuration is based on a direct SuperPro Designer simulation at this scale, consistent with the other 23 process configurations evaluated in this study. This result was cross-checked against a six-tenths power-rule estimate scaled from the corresponding 1000 L simulation (Peters et al. 2003). Following the standard six-tenths power-rule relationship: $\:{Cost}_{2}=\:{Cost}_{1}\times\:{\left(\frac{{Capacity}_{2}}{{Capacity}_{1}}\right)}^{0.6},\:$where Cost₁ and Capacity₁ correspond to the 1000 L simulation and Cost₂ and Capacity₂ to the 5000 L target scale.

#### Scenarios

All scenarios were structured in the flowsheet in preprocessing, upstream fermentation, and downstream processing sections, differing only in the preprocessing stage.

##### Scenario A: Commercial glucose (control)

The first scenario was modelled creating pure synthetic glucose, requiring no upstream preprocessing. Commercial glucose solution was prepared, heat sterilised and fed directly to the fermenters. No preprocessing capital is required. This scenario represents the current standard for *P. pastoris* fermentation media and serves as the economic reference against which waste-derived scenarios are compared.

##### Scenario B: Mechanically pressed fruit juice

Whole fruit biomass undergoes four-step mechanical pretreatment: solids storage (SL-101), shredding (SR-101), size reduction by grinding (GR-101), and solid-liquid extraction by leaching soluble components into an aqueous phase (SMSX-101). The resulting fruit juice stream (Feedstock S-101; glucose = 25.33 g L⁻¹) is stored before heat-sterilisation and fermenter feeding. Pomace is removed as solid waste. In the model, leaching represents 80% recovery of soluble sugars into the liquid fraction and a 10% loss of solvent to solids, values chosen within the typical industrial range for fruit pressing. The initial raw fruit composition was reconstructed in SuperPro Designer to match the content provided by the supplier (AINIA, Spain) so that leaching would yield the measured concentrations. Continuous chemostat operation for this scenario was simulated using a model projection calibrated at the same dilution rate (D = 0.05 h⁻¹) as Scenarios A and C, applying batch stoichiometry as no experimental chemostat data were available for this feedstock. While applying batch stoichiometry to the continuous projection of Scenario B represents a methodological limitation, it serves as a conservative economic baseline, ensuring that operational advantages are not overestimated in the absence of steady-state kinetic parameters for raw juice.

##### Scenario C: Enzymatically hydrolysed and nanofiltered (NF) sugar stream

The third scenario corresponded to a more intensive biorefinery-like route, in which fruit residues were mechanically pretreated and subsequently subjected to enzymatic hydrolysis and nanofiltration, conceptually consistent with lignocellulosic biowaste valorisation schemes (Eggeman and Elander 2005; Misailidis and Petrides 2020).

Fruit biomass undergoes the same mechanical pretreatment as Scenario B, followed by enzymatic saccharification in a cell culture reactor (BR-101) with added cellulolytic enzymes, and nanofiltration (UF-101) to concentrate the sugar stream and remove colloidal solids, enzyme residues, and fermentation inhibitors. The resulting NF feedstock contained a higher glucose concentration (30.51 g L^− 1^) than the mechanically pressed fruit juice (25.33 g L^− 1^), despite displaying a lower total sugar concentration ( 68.04 g L^− 1^ vs. 78.05 g L^− 1^). This scenario was therefore treated as a membrane-conditioned, enzymatically hydrolysed NF sugar stream rather than simply a higher-total-sugar concentrate.

In the SuperPro Designer flowsheet, this processing route incorporates additional equipment capital costs (BR-101 and UF-101) as well as operational expenditure associated with cellulolytic enzyme consumables and filtration energy inputs. In accordance with EU Directive 2018/851, this conditioned substrate is classified as a secondary raw material and assigned a positive acquisition cost in the economic model, reflecting the upstream processing intensity required prior to fermentation.

Enzymatic hydrolysis of cellulose and hemicellulose was modelled with a fixed conversion efficiency of 90%, and nanofiltration was assumed to retain solids while preserving soluble sugars in the permeate, conceptually aligned with published lignocellulosic biorefinery case studies (Eggeman and Elander 2005; Misailidis and Petrides 2020). In both waste scenarios, nitrogen associated with the waste was quantified and represented less than 5% of total nitrogen demand; it was therefore neglected in the stoichiometric nitrogen balance, and nitrogen was supplied as inorganic ammonium salts in the defined medium (Ferreira et al. 2018).

#### Stoichiometric modelling

Stoichiometric modelling of *P. pastoris* growth and CRL1 formation was performed in parallel with the experimental work to support process simulation in SuperPro Designer. Biomass was represented using the empirical formula CH₁.₈O₀.₅N₀.₂, consistent with standard elemental compositions for aerobic yeast biomass. A global aerobic growth reaction on glucose was defined as:

$$\begin{aligned} & {\mathrm{C}}_{{\mathrm{6}}} {\mathrm{H}}_{{{\mathrm{12}}}} {\mathrm{O}}_{{\mathrm{6}}} + {\mathrm{bO}}_{{\mathrm{2}}} + {\mathrm{cNH}}_{{\mathrm{3}}} \to {\mathrm{dCH}}_{{{\mathrm{1}}{\mathrm{.8}}}} {\mathrm{O}}_{{0.5}} {\mathrm{N}}_{{0.2}} \\ & \quad + {\mathrm{eCO}}_{{\mathrm{2}}} + {\mathrm{fH}}_{{\mathrm{2}}} {\mathrm{O}}. \\ \end{aligned}$$

Stoichiometric coefficients were determined by elemental mass balance and expressed as mass ratios, consistent with the SuperPro Designer reaction convention. CRL1 was modelled as a secreted extracellular product formed in parallel with biomass, using experimentally derived product-to-biomass ratios from the 0.75 L bench-scale data. CRL1 formation was treated as a parallel product term and did not modify the empirical biomass composition. Nitrogen naturally present in the biowaste-derived streams represented less than 5% of total nitrogen demand and was excluded from the stoichiometric equations; all nitrogen was supplied as inorganic ammonium salts (NH₄OH and (NH₄)₂HPO₄) in the defined mineral medium.

For continuous chemostat operation, distinct stoichiometric reactions were derived independently from batch reactions for Scenarios A and C, reflecting the different biomass and protein yields observed experimentally at steady state (D = 0.05 h⁻¹). The use of batch stoichiometry for continuous simulation would introduce systematic error given the marked differences in Y_X/S_ between modes (Scenario A: 0.114 batch vs. 0.515 chemostat; Scenario C: 0.342 batch vs. 0.580 chemostat. Table 4). For Scenario C, the stoichiometric model used Y_X/S_ = 0.580 g g⁻¹, which represents the yield on the sugar carbon fraction only. The experimentally determined value of 0.838 g g⁻¹ (Table 4) reflects additional biomass supported by assimilable organic nitrogen present in the NF stream (PAN 0.537 g L⁻¹; NH₄⁺ 0.106 g L⁻¹), consistent with the known effect of organic nitrogen sources on apparent biomass yield in (Martínez-Moreno et al. 2012). The full derivation of the corrected yield is provided in Supplementary Material S1.

**Scenario A Commercial glucose**

Batch: 30 Glucose + 9.46 O₂ → 16.665 Biomass + 1.213 Protein + 14.49 CO₂ + 7.092 H₂O

Glucose conversion was set to 99%, consistent with observed partial substrate utilisation in batch fermentation. The stoichiometric coefficient for biomass in the batch reaction (Y_X/S_ = 0.555 g g⁻¹) was derived from the 7 L scale-up experiment rather than the 0.75 L bench-scale run, where dissolved oxygen limitation during the exponential phase resulted in a lower apparent yield (0.114 g g⁻¹). The 7 L value is more representative of the oxygen transfer conditions achievable at larger scale. The maximum specific growth rate was set to µ_max_ = 0.153 h⁻¹.

*Chemostat* (*D * = 0.05 h⁻¹):

30 Glucose + 9.46 O₂ → 15.3 Biomass + 0.72 Protein + 14.96 CO₂ + 8.62 H₂O.

Glucose conversion was assumed at 99% in the stoichiometric model, consistent with near-complete substrate utilisation expected at D = 0.05 h⁻¹; as noted above, this reflects a modelling assumption rather than a directly measured conversion efficiency. This yields Y_X/S_ = 0.51 g DCW g⁻¹ glucose and Y_P/S_ = 0.024 g protein g⁻¹ glucose, consistent with the experimentally determined values at 0.75 L chemostat steady state (186 h, D = 0.05 h⁻¹).

**Scenario B: Mechanically pressed fruit juice**

*Batch only (no continuous experimental data available)*:

Two parallel reactions for glucose and fructose were implemented at *µ*_*max*_ = 0.127 h⁻¹ and 100% conversion was assumed:

30 Glucose + 9.46 O₂ → 13.200 Biomass + 3.063 Protein + 4.77 CO₂ + 18.427 H₂O (Y_x/s_ = 0.445 g. g⁻¹).

30 Fructose + 9.46 O₂ → 13.200 Biomass + 3.063 Protein + 4.77 CO₂ + 18.427 H₂O (Y_x/s_ = 0.445 g. g⁻¹).

The identical stoichiometric coefficients for glucose and fructose reflect their equivalent metabolic fate following phosphorylation in *P. pastoris* (García-Ortega et al. 2019). For chemostat simulation, batch stoichiometry was applied as a conservative projection in the absence of experimental continuous cultivation data for this feedstock.

**Scenario C: Enzymatically hydrolysed NF sugar stream**

Batch: Two parallel reactions for glucose and fructose were implemented at µ_max_ = 0.127 h⁻¹ and 100% conversion was assumed in the stoichiometric model:

30 Glucose + 9.46 O₂ → 13.200 Biomass + 5.204 Protein + 4.77 CO₂ + 16.286 H₂O (*Y*_X/S_ = 0.445 g. g⁻¹).

30 Fructose + 9.46 O₂ → 13.200 Biomass + 5.204 Protein + 4.77 CO₂ + 16.286 H₂O (*Y*_X/S_ = 0.445 g. g⁻¹).

*Chemostat *(*D* = 0.05 h⁻¹):

30 Substrate + 9.6 O₂ → 17.40 Biomass + 3.30 Protein + 14.96 CO₂ + 3.94 H₂O.

Substrate conversion was assumed at 100% in the stoichiometric model, This yields Y_X/S_ = 0.58 g DCW g⁻¹ substrate and Y_P/S_ = 0.110 g protein g⁻¹ substrate (D = 0.05 h⁻¹).

#### Scale up simulation in SuperPro designer

Bench-scale aeration was set at 1.5 vvm (1.125 L min⁻¹ at 0.75 L working volume). Scale-up followed constant P/V (236 W m⁻³) and constant superficial gas velocity (vs. = 3.5 × 10⁻³ m s⁻¹); where both could not be maintained simultaneously, P/V was prioritised. Full hydrodynamic equivalence across scales was not assumed, as mixing time and local oxygen gradients change with vessel size (Doran 2012).

A base case was first developed at 0.75 L working volume for each scenario in batch and batch-to-chemostat modes, correlated to reproduce kinetic and yield obtained experimentally. The model was then adapted to the fruit juice and enzymatically hydrolysed NF sugar stream scenarios by replacing the carbon stream, stoichiometric equations and inserting the corresponding preprocessing steps. Each of the three feedstocks was simulated in batch followed by continuous chemostat modes at four scales: 1 L, 100 L, 1000 L and 5000 L working volume.

Upstream batch fermentations consisted of media preparation, inoculum preparation, and a batch fermenter. Fermentation was described by a Monod model, which is widely used for aerobic microbial growth (Doran 2012), with glucose and fructose treated as substrates of identical kinetic behaviour. The maximum specific growth rate µ_max_ was adjusted for each scenario to reproduce the 0.75 L experimental biomass profiles, whereas the affinity constant K_s_ and stoichiometric yields were kept constant for comparability. Fermentation temperature and pH were fixed at 25 °C and 4.7, matching experimental conditions and eliminating the need for additional pH conditioning before downstream processing. Continuous chemostat operation was represented by extending the batch simulation. After the batch phase, the fermenter was switched to continuous feeding with SUBS containing 50 g L⁻¹ total sugars at a dilution rate of 0.05 h⁻¹, with equal outlet flow to maintain constant volume and a residence time of 20 h.

Downstream processing in the model was represented by conditioning, centrifugation and membrane-based concentration operations that approximated the experimental centrifugation and TFF workflow using available SuperPro unit operations (Ferreira and Petrides 2020; Colacicco et al. 2024). The fermentation broth was assumed to enter DSP at pH 4.7 and was cooled to 15 °C prior to clarification. Biomass was removed by disk-stack centrifugation (DS-101; 99% biomass removal), with CRL1 assumed to remain in the clarified supernatant. The clarified broth was then concentrated by ultrafiltration/TFF, represented in SuperPro using the closest available membrane-separation unit operation. A concentration factor of 5 was assumed, with 80% overall CRL1 recovery to account for losses during clarification and concentration. This value was adopted as a conservative estimate at the time of model development, prior to completion of the 7 L TFF trial (Sect. 3.2.2), which subsequently achieved an experimental concentration factor of 8.7× under comparable conditions. Retaining the lower, more conservative value across all 24 process configurations avoids overestimating downstream performance and provides a cautious basis for the reported economic outcomes; if anything, this suggests that the unit production costs reported here may be modestly overestimated relative to what could be achieved using the experimentally validated concentration factor. Clean-in-place sequences consisting of water and 2% NaOH rinses were incorporated for both the bioreactor and the TFF system after each operational cycle.

**Fig. 1:**
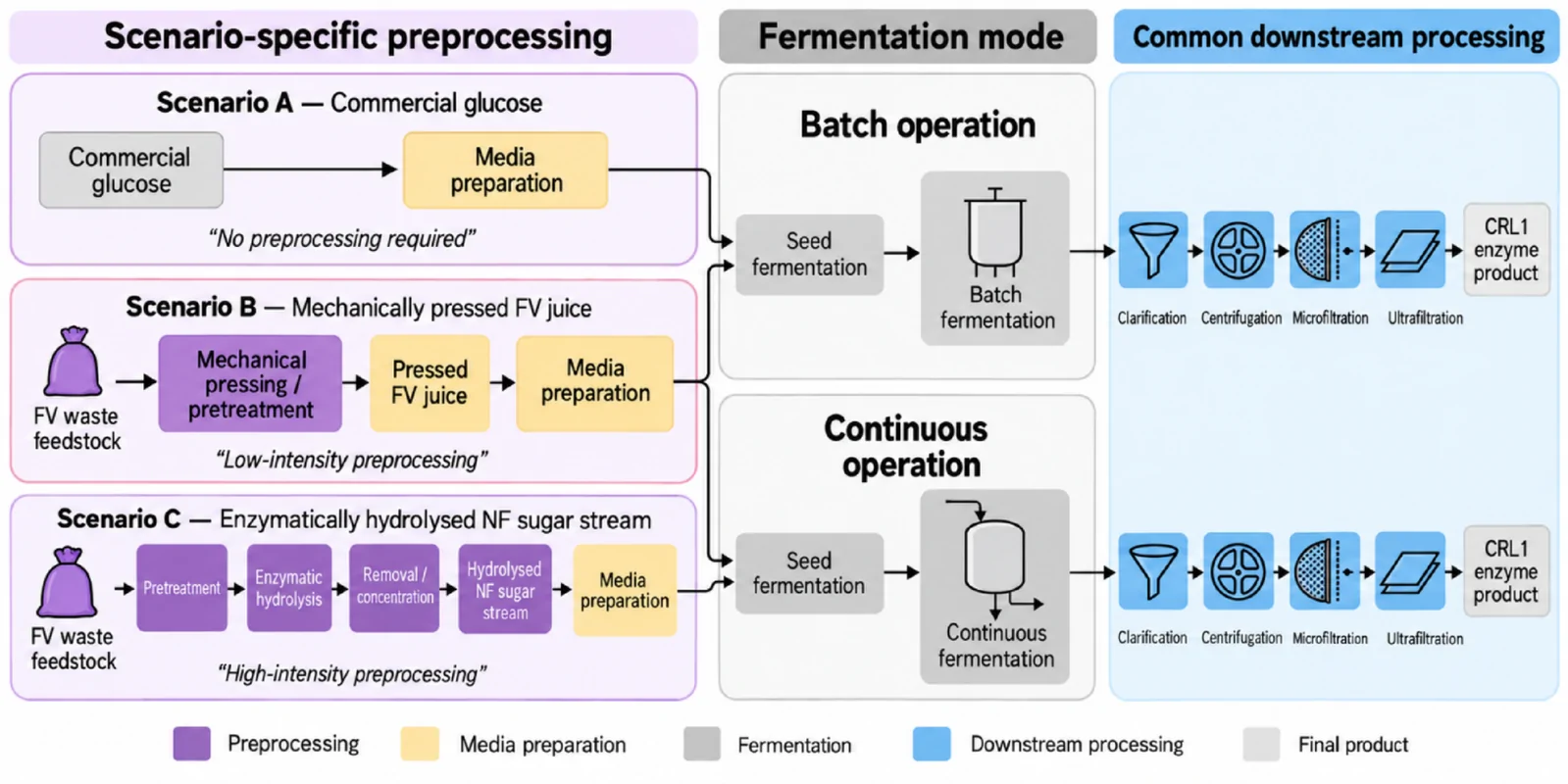
SuperPro Designer v14 process flowsheet showing the four main sections of the CRL1 production process: preprocessing (Scenarios B and C only), media preparation, fermentation (batch and chemostat modes) and downstream processing. Scenario A omits the preprocessing section. The complete process flowsheets for all three scenarios at 5000 L continuous mode are provided as Supplementary Figures S1–S3

For each target scale, a separate SuperPro Designer project was created with the fermenter vessel volume set to 1, 100, 1000 and 5000 L and 75% its corresponding working volume. Residence times in heat-sterilisation units, centrifuges and microfiltration were adjusted so that resulting equipment sizes and throughputs fell within published industrial ranges for each unit operation class (Ferreira and Petrides 2020). The process flowsheet comprised four sections: preprocessing, media preparation, fermentation, and downstream processing, as shown in Fig. 1.

### Technoeconomic analysis

The techno-economic analysis was conducted in SuperPro Designer (Ferreira and Petrides 2020); Intelligen, 2019). For each scenario, operating mode (batch or continuous) and scale, an Economic Evaluation Report (EER) was generated (Supplementary material). Main indicators included capital expenditure (CAPEX), operating expenditure (OPEX), annual revenues, unit production cost (UPC), return on investment (ROI), and payback time. Although reports were generated at all scales, quantitative economic interpretation focused on the 5000 L scale, for which the cost correlations are most appropriate; values at 1 L and 100 L were treated as indicative.

To ensure modeling consistency across all twenty-four process configurations, key operational, utility, and labor cost frameworks were standardized based on industrial baseline data for Spain, and Inflation-adjusted uniformly to 2025 US Dollars (USD). The complete set of economic parameters, technical justifications, and foundational sources applied across the simulations is detailed in Table 3. The biorefinery plant lifetime was modelled across a 15-year economic horizon with a project Weighted Average Cost of Capital (WACC) fixed at 7.0%, using SuperPro Designer default settings (Ferreira and Petrides 2020). Labor cost structures were derived from Eurostat (Eurostat 2025) industrial compensation databases for Spain, applying a conservative 1.8× burden multiplier to the base industrial hourly rate to integrate direct wages, compulsory social contributions, administrative supervision, and plant overheads, yielding a loaded labor rate of $54.74/h. The annual operating time (AOT) was bounded at 7920 h/yr, representing a 330-day continuous operational window with a 35-day annual allocation for scheduled maintenance and clean-in-place (CIP) protocols. Primary downstream processing (DSP) recovery was parameterised at an 80% cumulative yield, factoring in sequential industrial disk-stack centrifugation followed by tangential-flow ultrafiltration (TFF-UF) concentration modules. All economic inputs were Inflation-adjusted and are expressed uniformly in 2025 US Dollars (USD) pricing matrices.

**Table 3:** Economic parameters and assumptions applied uniformly across all twenty-four process configurations. WACC = Weighted Average Cost of Capital; TPC = Total Plant Cost; DFC = Direct Fixed Capital; DSP = Downstream Processing; NF = Nanofiltration; TFF-UF = Tangential-Flow Filtration Ultrafiltration

Parameter	Value	Technical justification/source
Cost basis year	2025	Intelligen Inc. SuperPro Designer v14 Inflation-adjusted from *SuperPro Designer* defaults
Discount rate (WACC)	7.0%	Standard bioprocess TEA benchmark (Humbird et al. 2011)
Plant lifetime	15 years	Standard capital depreciation (Ferreira et al. 2018)
Annual operating time	7920 h yr⁻¹ (330 days)	330 operational days yr⁻¹ (Cunniffe et al. 2025)
DSP Unit Operations & Yield	80% (cumulative)	Disk-Stack Centrifugation + TFF-Ultrafiltration (15 kDa)
CRL1 bulk selling price	$25 kg⁻¹ (base case)	Low-range unpurified commodity lipase benchmark (Ferreira et al. 2018; Chandra et al. 2020)
Commercial glucose (Scenario A)	$0.70 kg⁻¹	Bulk industrial dextrose syrup pricing (Humbird et al. 2011).
Crude Waste Fruit (Scenario B)	$0.00 kg⁻¹	Zero-cost gate-fee model; zero material acquisition penalty
Scenario C Intermediate Stream)	$0.00 kg⁻¹	Modelled internally via upstream enzymatic hydrolysis and NF CAPEX
Loaded Labor rate	$54.74 h⁻¹	Eurostat industrial baseline with a 1.8× overhead/social burden factor(Eurostat 2025)
Industrial Electricity	$0.13 kWh⁻¹	Non-household electricity price for Spain, medium-size industrial consumers (€0.12 kWh⁻¹); converted at EUR/USD = 1.08 (ECB average 2024). Spain’s industrial electricity is approximately 20% below the EU average due to renewable energy deployment (Eurostat 2026)
Process Steam (High Pressure)	$32.00 MT⁻¹	Standard utility generation matrix (Ferreira and Petrides 2020)
Process/Fermentation Water	$1.85/MT	Purified industrial supply tariff required for Scenario B washing blocks. (Ferreira and Petrides 2020)
Project Contingency Factor	15% TPC/5% DFC	Standard greenfield site capital buffer (Ferreira and Petrides 2020)

### Sensitivity and break-even analysis

A local, one-at-a-time (OAT) sensitivity analysis was performed for the 5000 L commercial-scale configurations to identify the cost drivers with the greatest influence on the unit production cost (UPC, $ kg⁻¹ CRL1) (Ferreira et al. 2018). Seven parameters were varied independently over a range of ± 50% relative to their baseline values whereas all other variables were held constant, following standard TEA practice (Eggeman and Elander 2005; Ferreira et al. 2018; Cunniffe et al. 2025). The parameters evaluated included: CRL1 selling price, biowaste feedstock acquisition cost, enzymatic hydrolysis efficiency, final secreted CRL1 titre, annual operating time, downstream processing recovery, and for continuous configurations dilution rate. Changes in UPC relative to the baseline were used to rank parameters by economic influence, visualised as tornado plots.

The minimum product titre required for economic viability defined as the break-even titre (BET, g L⁻¹) at which UPC equals the commodity lipase reference price of USD $25 kg⁻¹. BET was calculated independently for batch and continuous operating modes.

For batch operation, BET was derived as:

$$\:{BET}_{batch}\:\left[g\:{L}^{-1\:}\right]=\frac{Annual\:cost}{{V}_{Ferm\:\left[L\right]}\:\times\:\:{Y}_{DSP}\times\:\:{N}_{batches}\:\times\:\mathrm{P}\mathrm{r}\mathrm{i}\mathrm{c}\mathrm{e}\:\mathrm{C}\mathrm{R}\mathrm{L}1\:\times\:\:0.001}$$

For continuous chemostat operation, the discrete batch frequency term is replaced by the steady-state throughput defined by the dilution rate and annual operating time:

$$\:{BET}_{Continuous}\:\left[g\:{L}^{-1\:}\right]=\frac{Annual\:\mathrm{c}\mathrm{o}\mathrm{s}\mathrm{t}}{{V}_{Ferm\:\left[L\right]}\:\times\:\:{Y}_{DSP}\times\:AOT\:\times\:D\:\:\times\:Price\:CRL1\:\times\:0.001}$$

where V_Ferm_ is fermenter working volume (L), Y_DSP_ is the downstream processing recovery factor (0.80), N_batches_ is the maximum number of annual batches per year, AOT is annual operating time (7920 h yr⁻¹), D is dilution rate (0.05 h⁻¹), price is the reference CRL1 selling price ($ kg⁻¹), and 0.001 is the dimensional consistency factor converting grams to kilograms.

Annual Cost is the annual operating cost (OPEX) as reported by SuperPro Designer, which includes facility-dependent charges comprising straight-line capital depreciation over a 15-year plant lifetime, maintenance, insurance, and factory expense. No separate capital annualisation was applied, as depreciation is already embedded within the facility-dependent cost category.

Economic viability was defined as UPC ≤ USD $25 kg⁻¹ combined with a positive net present value (NPV > 0) over the 15-year plant lifetime (Peters et al. 2003). Sensitivity results were processed and visualised in Python v3.10 using the pandas and matplotlib libraries.

##  Results and discussion

Table 4 consolidates all experimental fermentation performance across the three carbon-source scenarios, operating modes, and all working volumes evaluated.

**Table 4:** Consolidated experimental fermentation performance across all carbon-source scenarios, operating modes and working volumes

Mode/time	Sampling time (h)	Vol (L)	DCW (g.L^-1^)	Y_X/S_ (g.g^-1^)	Protein BCA (g. L^-1^)	Enzymatic Activity (U. L^-1^)	Vol. Prod. (U.L.h^-1^)	Sp. Prod. (U.g.h^-1^)
*Scenario A — Commercial Glucose*								
Batch	24	0.75	4.57	0.114	2.37	0.16	0.006	0.001
Chemostat D = 0.05 h^-1^	186	0.75	25.75	0.515	1.20	1.69	0.084	0.003
*Scenario B — Mechanically Pressed FV Juice*								
Batch	23	0.75	5.40	0.135	7.77	3.79	0.16	0.030
Batch	23	7	27.83	0.695	4.23	1.87	0.08	0.003
*Scenario C — Enzymatically Hydrolysed NF Sugar Stream*								
Batch	40	0.75	13.7	0.342	8.81	2.71	0.07	0.005
Chemostat D = 0.05 h^-1^	158	0.75	41.9	0.838	5.51	0.41	0.02	0.0005

Vol. Prod. (volumetric productivity): batch = activity ÷ fermentation time; chemostat = activity × D. Sp. Prod. (specific productivity) = Vol. Prod. ÷ DCW. Yx/s = DCW ÷ S₀ (batch S₀ = 40 g L⁻¹; chemostat feed S₀ = 50 g L⁻¹). Elevated Y_X/S_ for Scenario C chemostat reflects the presence of supplementary organic nitrogen in the NF stream (PAN 0.537 g L⁻¹; NH₄⁺ 0.106 g L⁻¹), which contributed to biomass accumulation beyond the calculated sugar-fraction yield. All runs pH 4.7, 25 °C, *n* = 1

**Fig. 2:**
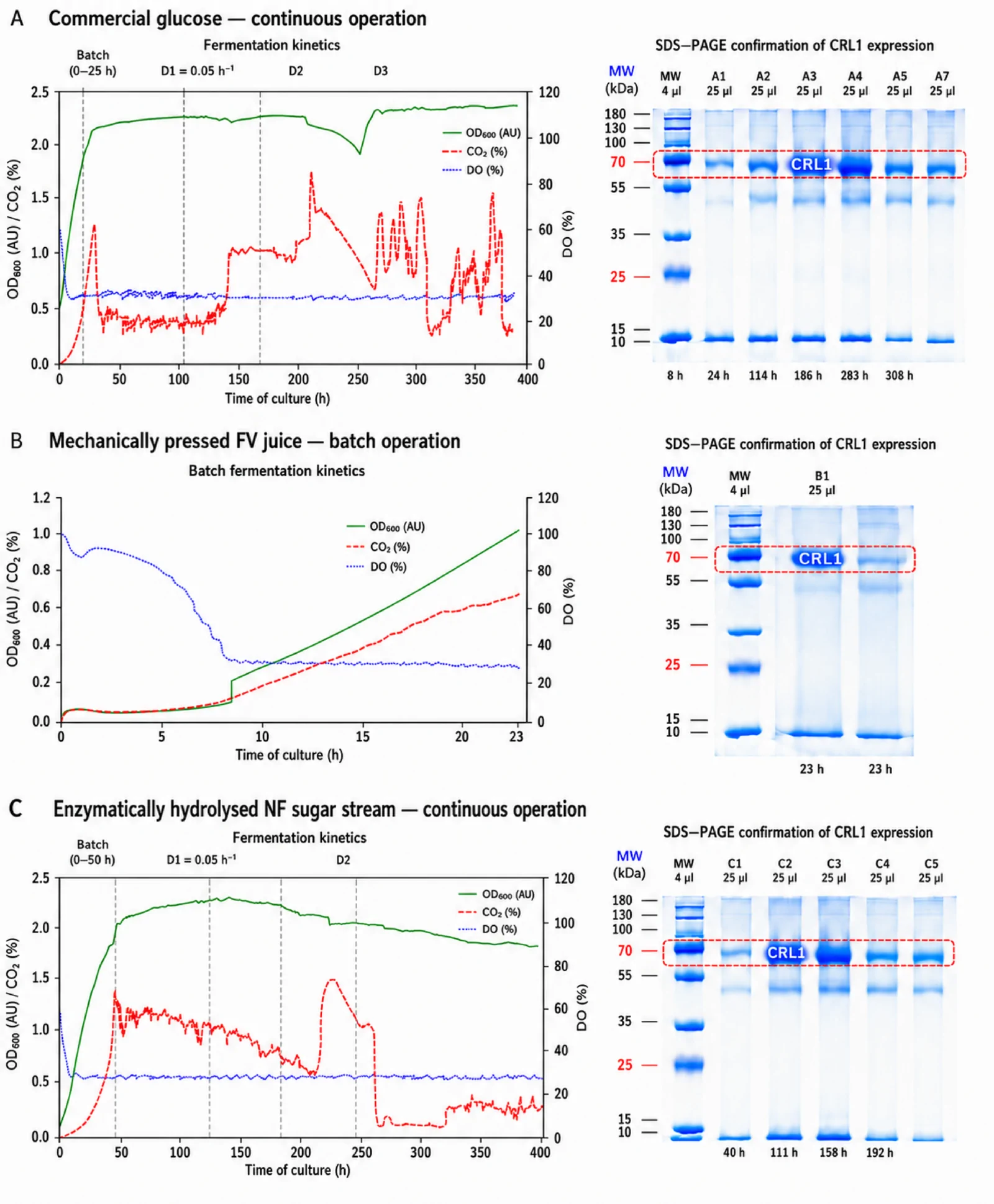
Online fermentation kinetics (left panels) and SDS–PAGE gels of clarified supernatant (right panels) for the three carbon-source scenarios. Kinetics: CO₂ (%), OD₈₆₀ (CU) and DO (%) recorded continuously by ROSITA^®^ software (Bionet, Spain); vertical dashed lines indicate phase transitions. Gels: MW markers (4 µL) in the first lane; all sample lanes at 25 µL; 70 kDa CRL1 band indicated by a red dashed box. **A** Commercial glucose. Lanes A1–A7: 8 h, 24 h (batch end), 114 h, 186 h (chemostat, D = 0.05〖” h” 〗^(-1)), 283 h, 308 h. **B** Mechanically pressed FV juice. Lane B1: 0.75 L batch, 23 h. Lane B2: 7 L batch, 23 h. **C** Enzymatically hydrolysed NF sugar stream. Lanes C1–C5: 40 h (batch end), 111 h, 158 h (chemostat, D = 0.05 h⁻¹)

Protein concentration was determined by BCA assay throughout, measuring total extracellular protein. Bradford assay systematically underestimated values across all scenarios due to matrix interference and is reported in Table 4 for completeness only (Kruger 1994; Reichelt et al. 2016). SDS–PAGE confirmed CRL1 expression qualitatively at 70 kDa (Fig. 2).

### Scenario A: glucose control

#### Batch fermentation

Batch fermentation with glucose as the defined carbon source was conducted at 0.75 L with an initial glucose concentration of 40 g L⁻¹ at pH 4.7 and 25 °C. Biomass reached 4.57 g DCW L⁻¹ at 24 h with Yx/s = 0.114 g g⁻¹ glucose (Table 4). The lower Yx/s relative to the chemostat (0.515 g g⁻¹) may be associated with oxygen-transfer limitation during the exponential batch phase, where the agitation-aeration cascade (300–1,800 rpm, 1.5 vvm) operated near its upper limit. Under such conditions, *P. pastoris* may shift from fully respiratory metabolism towards a respiro-fermentative state, reducing biomass yield and potentially favouring by-product formation. Lipase activity at 24 h was 0.16 U L⁻¹, giving a volumetric productivity of 0.006 U L⁻¹ h⁻¹. SDS–PAGE (Fig. 2A, lane A2) confirmed CRL1 expression at 70 kDa, consistent with N-glycosylation of the 60 kDa unglycosylated backbone during secretion through the *P. pastoris* secretory pathway (García-Ortega et al. 2019).

#### Continuous cultivation—dilution rate screening and steady state

Three dilution rates were evaluated sequentially within a single continuous cultivation: D = 0.025, 0.040 and 0.050 h⁻¹, corresponding to residence times of 40, 25 and 20 h, respectively; D = 0.05 h⁻¹ was confirmed as the operational steady state for CRL1 production (Fig. 2A). D1 = 0.05 h⁻¹ (30–130 h) yielded stable OD₈₆₀ (~ 2.1 CU) and exhaust CO₂ (0.60%), confirmed within ± 5% over five consecutive residence times, achieving steady state. Increasing the dilution rate at D2 = 0.06 h⁻¹ (131–231 h) elevated CO₂ output and caused OD instability; reducing it at D3 = 0.07 h⁻¹ (232–300 h) produced large CO₂ spikes and OD oscillation without re-establishing steady state. D = 0.05 h⁻¹ was therefore selected as the operating condition for TEA simulation.

Chemostat operation at D = 0.05 h⁻¹ increased biomass relative to batch (25.75 vs. 4.57 g DCW L⁻¹) and substrate-to-biomass conversion (*Y*_x/s_ 0.515 vs. 0.114 g DCW g⁻¹ glucose). BCA protein at steady state was 1.20 g L⁻¹. Enzymatic activity increased from 0.16 U L⁻¹ in batch to 1.69 U L⁻¹ in chemostat; volumetric productivity (enzymatic activity × D) was 0.08 U L⁻¹ h⁻¹—a 12.6-fold improvement over batch (0.006 U L⁻¹ h⁻¹). This productivity gain at sustained throughput provides the primary technical justification for continuous operation in the TEA, consistent with findings from comparable recombinant enzyme TEA studies where continuous operation reduced unit production cost relative to batch (Ferreira and Petrides 2020; Cunniffe et al. 2025).

### Scenario B: mechanically pressed fruit and vegetable juice

#### Batch fermentation

*P. pastoris* was cultivated on mechanically pressed fruit and vegetable (FV) juice (Table 2) at 0.75 L and 7 L working volume with an initial total sugar concentration of 40 g L⁻¹ at pH 4.7 and 25 °C. OD₈₆₀ increased continuously throughout the run (Fig. 2B), consistent with a µ_max_ of 0.127 h⁻¹. Biomass reached 5.40 and 27.83 g DCW L⁻¹ at 0.75 L and 7 L respectively, with Yx/s increasing from 0.135 to 0.695 g g⁻¹ total sugars. The apparent Yx/s at 7 L exceeds the theoretical maximum on hexoses (~ 0.51 g g⁻¹), consistent with assimilation of organic nitrogen compounds present in the FV juice matrix, including free amino acids and primary amino nitrogen,, which can also serve as supplementary carbon sources during cultivation (Martínez-Moreno et al. 2012). Beyond the quantified total sugars, the biowaste matrix contains significant amounts of non-sugar carbon sources, such as organic acids (mainly malic and citric acids) and soluble nitrogenous compounds (amino acids and peptides). Under aerobic conditions, *P. pastoris* is capable of co-assimilating these organic molecules through active transport systems and channelling them directly into the tricarboxylic acid (TCA) cycle or central metabolism (Mattanovich et al. 2009). Consequently, these alternative carbon fractions contributed substantially to biomass accumulation, artificially inflating the yield calculated solely on a total sugar basis.

*P. pastoris* in this scenario achieved the highest volumetric CRL1 productivity of all batch conditions (0.165 U L⁻¹ h⁻¹ at 0.75 L), indicating that *pGAP*-driven CRL1 secretion is well-supported by the fructose-glucose mixture of the crude FV matrix during active growth. SDS–PAGE (Fig. 2B) confirmed CRL1 expression at 70 kDa at both scales. Additional lower-molecular-weight bands indicate co-secretion of non-target proteins from the complex FV juice matrix. Continuous fermentation on FV juice was not evaluated experimentally due to feedstock limitation after scaling up tests at 7 L; the Scenario B chemostat model is a projection based on batch stoichiometry at D = 0.05 h⁻¹ and is interpreted separately with higher uncertainty. Experimental chemostat validation on mechanically pressed FV juice is a priority for further work.

#### Downstream processing—TFF concentration (7 L batch)

Downstream processing was evaluated on clarified supernatant from the 7 L FV juice batch. Centrifugation yielded approximately 4 L of clarified supernatant; subsequent tangential-flow filtration (TFF) with a 15 kDa nominal molecular weight cut-off ceramic membrane achieved an 8.7× concentration factor against a theoretical target of 13× (Table 5). This fouling burden is expected to increase in absolute terms at industrial scale, since resistive layer formation scales with the total membrane area required to process a given throughput; the specific flux decline per unit area, however, may not scale linearly if transmembrane pressure and cross-flow velocity are re-optimised (de Barros et al. 2003).

The 8.7-fold concentration factor achieved fell short of the theoretical target of 13-fold due to progressive fouling of the ceramic membrane by residual pectin, consistent with the known formation of resistive gel and pore-blocking layers by pectic polysaccharides on ultrafiltration membranes (de Barros et al. 2003; Ruby Figueroa et al. 2011; Parvarei 2022). Three mitigation strategies follow: enzymatic depectinisation of the feed prior to filtration, which the juice processed here did not undergo; more aggressive upstream solids removal, already implemented via the centrifugation stage added to the flowsheet in response to this fouling event; and periodic membrane backflushing and chemical cleaning, already included in the model as clean-in-place sequences, whose frequency may need to increase at scale.

**Table 5:** Tangential-flow filtration performance: 7 L FV juice batch. Single run (*n* = 1). 15 kDa nominal molecular weight cut-off ceramic membrane. Theoretical concentration factor 13×; achieved 8.7×. Protein by BCA assay. Activity by chromogenic lipase assay, TFF pre-filter clogged by residual pectin; centrifugation used for primary clarification

Stream	Protein BCA (g L^–1^)	Activity (U L^–1^)	Notes
Feed (post-centrifugation)	0.16	0.78	4 L clarified FV juice supernatant (7 L batch)
Retentate	1.39	1.83	8.7× concentration achieved; theoretical 13×; CRL1 dominant band (70 kDa) by SDS–PAGE
Permeate	0.01	0.014	Partial CRL1 passage through 15 kDa ceramic membrane confirmed by activity

During operation, the TFF pre-filter clogged and permeate flux declined rapidly, attributed to soluble and colloidal pectin in the raw FV juice. Pectin is documented to form resistive gel layers on ultrafiltration membrane surfaces under pressure-driven flow (Ruby Figueroa et al. 2011; Parvarei 2022). In the SuperPro Designer flowsheet, a disk-stack centrifugation stage was therefore included upstream of the membrane modules to remove suspended solids and colloidal pectin prior to filtration. Bench-scale validation of continuous operation on FV juice, including fouling dynamics under steady-state conditions, is required before the scale-up assumptions can be confirmed.

### Scenario C: enzymatically hydrolysed NF sugar stream

#### Batch versus continuous cultivation

*P. pastoris* was cultivated on the enzymatically hydrolysed NF sugar stream (glucose 30.51 g L⁻¹, fructose 35.28 g L⁻¹; NH₄⁺ 0.106 g L⁻¹; primary amino nitrogen 0.537 g L⁻¹) under batch, chemostat at 0.75 L (*n* = 1). Batch fermentation (40 h) yielded 13.7 g DCW L⁻¹ with Y_X/S_ = 0.342 g g⁻¹ and the highest BCA protein of all batch conditions (8.81 g L⁻¹). SDS–PAGE (Fig. 2C) confirmed CRL1 expression at ~ 70 kDa.

Two dilution rates were evaluated sequentially. D = 0.05 h⁻¹ (50–150 h) achieved stable steady state confirmed by OD_860_ (~ 2.1 CU) and CO₂ (~ 0.75–1.00%) within ± 5% over five consecutive residence times. Increasing to a higher dilution rate D2 = 0.06 h⁻¹ (151–235 h) and D = 0.07 h⁻¹ (236–308 h) produced an initial CO₂ spike followed by a sharp decline to near-zero output (Fig. 2C), indicating metabolic disruption and failure to re-establish steady state on this substrate at higher flow rates. D = 0.05 h⁻¹ was therefore retained as the sole validated operating condition for the Scenario C simulation.

Chemostat steady state (D1 = 0.05 h⁻¹) achieved DCW = 41.9 g L⁻¹, BCA protein 5.51 g L⁻¹ and activity 0.41 U L⁻¹. The corresponding apparent biomass yield Y_X/S_ = 0.838 g g⁻¹ measured fermentable sugar. This value should be interpreted as an apparent yield based on the quantified glucose and fructose fraction, rather than as a yield on the total organic content of the NF stream. Because the NF stream is a complex waste-derived substrate, additional soluble components not captured in the measured sugar fraction, particularly assimilable organic nitrogen measured as PAN, may have contributed both nitrogen and carbon skeletons for biomass formation. Ammonium present in the stream may also have supported nitrogen assimilation, although it does not contribute carbon. For the SuperPro Designer stoichiometric model, a corrected Y_x/s_ of 0.580 g DCW g⁻¹ measured sugar was therefore used to represent biomass formation attributable to the sugar-carbon fraction only. The correction procedure is provided in Supplementary Material.

Despite achieving higher biomass than Scenario A, the Scenario C chemostat showed lower volumetric productivity than the corresponding batch fermentation (0.02 vs. 0.07 U L⁻¹ h⁻¹), in contrast to the 12.6-fold improvement observed for Scenario A. The reduced biomass-specific productivity on the NF stream may reflect the presence of inhibitory compounds introduced or concentrated during enzymatic hydrolysis and membrane processing, including organic acids and phenolic derivatives, which have been reported to affect metabolic activity in *P. pastoris* in a dose-dependent manner (Paes et al. 2021). Dilution-rate effects also cannot be excluded, as q_P_ under *pGAP* control is growth-rate dependent (Rebnegger et al. 2014), and the composition of the NF substrate composition may shift the optimal dilution rate relative to glucose. The inability to re-establish stable operation at higher dilution rates suggests that the operational window for this substrate may be narrower than for the defined glucose medium.

### Techno-economic evaluation: CAPEX, OPEX, and scaling dynamics

This analysis evaluates three carbon-source scenarios across batch and continuous operating modes at four scales, using experimental bench-scale data as the basis for process simulation. All kinetic and yield parameters were derived from bench-scale experiments at 0.75–3 L (*n* = 1 throughout). The continuous Scenario B flowsheet was developed as an in-silico projection derived from the experimental batch stoichiometry, establishing a predictive operational baseline in the absence of continuous-culture data for this specific substrate. Scale-up assumes constant P/V and vs., without experimental verification above bench scale. Published TEA studies for recombinant protein production in *P. pastoris* using SuperPro Designer are available for defined substrates under methanol-inducible promoters, including a recent comparison of fed-batch and continuous cultivation for KEX_2_ protease (Yamanie et al. 2026). To the best of the authors’ knowledge, no equivalent analysis has been identified for FV waste-derived substrates under constitutive *pGAP* expression, which limits direct benchmarking of the cost structures reported.

#### Interpreting process economics through the TRY framework

Titer, rate, and yield (TRY) are the three fermentation metrics that determine process economics, and each affects a different cost category (Konzock and Nielsen 2024). Understanding their individual effects is essential for interpreting the TEA outputs critically. Titer (secreted CRL1, g L⁻¹ BCA) determines the unit production cost directly (Konzock and Nielsen 2024). It is used as denominator of the UPC equation and governs downstream processing costs, which scale with the dilution factor required to reach the product concentration. At current experimental titres (1.2–8.8 g L⁻¹ BCA across all conditions and modes), the UPC remains 47 to 1442-fold above the commodity benchmark across all scenarios at the 5000 L commercial scale.

**Fig. 3:**
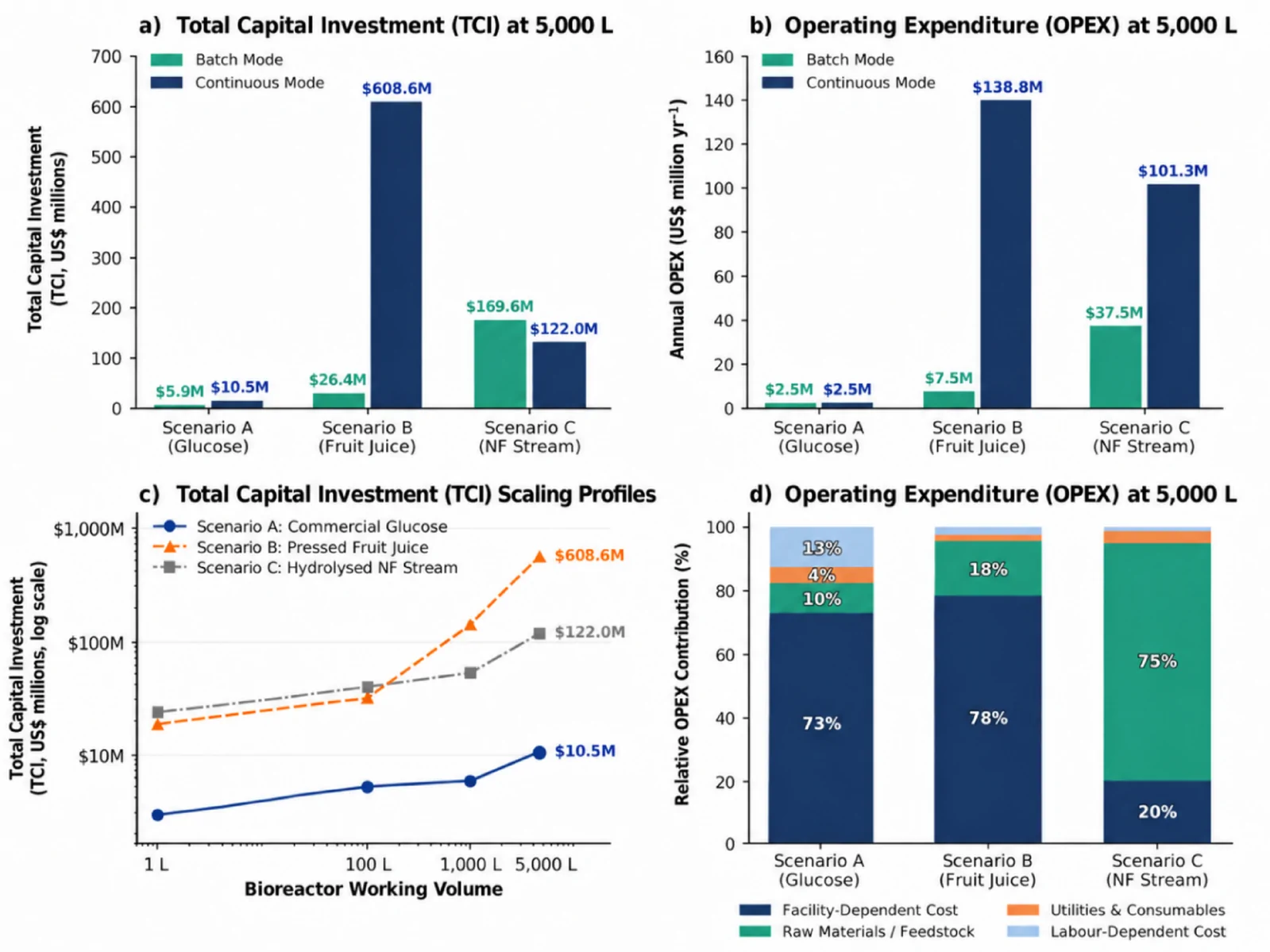
Comparative techno-economic analysis and capacity scaling profiles for recombinant CRL1 lipase production across three carbon-source scenarios. **a** Total Capital Investment (TCI) at 5000 L fermenter capacity, comparing batch and continuous chemostat operating modes. **b** Annual Operating Expenditure (OPEX) at 5000 L. **c** TCI scaling profiles from 1 to 5000 L under continuous operation. **d** Relative OPEX contribution breakdown under continuous operation at 5000 L. All values from SuperPro Designer v14 Economic Evaluation Reports (2025 USD). Scenario C batch 5000 L in panel (**a**) from direct simulation, cross-checked against the six-tenths power rule.

Rate (volumetric productivity, U L⁻¹ h⁻¹) determines reactor capital utilisation (Konzock and Nielsen 2024). Higher rate means more product from the same vessel at the same time, reducing facility-dependent cost per kg. Chemostat operation improved volumetric rate 12.6-fold over batch on glucose (0.08 vs. 0.006 U L⁻¹ h⁻¹), and this is the primary engineering justification for continuous operation in the TEA. Yield (*Y*_*X/S*_ and *Y*_*P/*S_, g g⁻¹) influences the amount of feedstock required per unit of product and therefore affects raw material-related costs. However, its economic relevance depends on the relative contribution of feedstock and processing reagents to the total OPEX (Konzock and Nielsen 2024). In the 5000 L continuous scenarios, raw material costs represented 9.7% of total OPEX in Scenario A and 18.0% in Scenario B, indicating that yield-related improvements would have a limited effect on overall cost reduction in these cases. By contrast, in Scenario C, raw material costs accounted for 75.1% of total OPEX, making yield and feedstock conversion efficiency more economically relevant (Fig. 3d).

For Scenarios A and B, these results indicate that substrate yield is not the main economic constraint, as raw material costs represent only a minor fraction of total OPEX. Scenario C is the exception, where enzymatic hydrolysis reagents dominate raw material-related costs and yield improvements would therefore have a measurable economic impact. This differentiated role of the TRY metrics is consistent with Konzock and Nielsen (2024), who note that for high-value, low-titre bioproducts, titre and rate tend to dominate over yield in determining cost competitiveness. The holistic comparative techno-economic analysis, capital profiles, and operational breakdowns reflecting these metrics across all scenarios are integrated and presented in Fig. 3.

Across all scenarios, TCI increases sub-linearly with scale (Fig. 3c), consistent with the six-tenths power rule for capital cost scaling in chemical engineering (Peters et al. 2003), and the economic comparison between scenarios becomes meaningful only at 1000 L and above.

The disproportionate TCI of $608.6 M for Scenario B at 5000 L highlights a structural constraint of single-flowsheet process integration(Aranda-Barradas et al. 2025). In the model developed, the preprocessing units are sised assuming the biorefinery absorbs the raw fruit processing in real-time. In an industrial ecosystem, this capital burden would be mitigated by decoupling feedstock preparation from the fermentation facility, sourcing the pressed juice from existing agricultural juice-processing plants under a centralised supply model, which would eliminate the need for upstream shredding and extraction capital at the bioprocess site. As a circular alternative, Poddar and Scown (2025) highlight that early-stage bioproduct facilities can reduce capital risk by outsourcing solid waste and wastewater management to specialist entities. The pomace stream generated in Scenarios B and C, the solid residue after mechanical pressing, is removed as waste in the current model without further valorisation. Its potential use as a soil amendment, animal feed, or compost feedstock could partially offset preprocessing costs in a more complete biorefinery configuration.

#### Capital expenditure CAPEX across scenarios and scales

Annual OPEX at 5000 L continuous is $2.55 M for Scenario A, $138.8 M for Scenario B, and $101.3 M for Scenario C (Table 7; Fig. 3b). In Scenarios A and B, facility-dependent costs such as capital depreciation, maintenance, insurance, and plant overhead, represent the dominant OPEX category (73.2% and 78.1% respectively, as broken down in Fig. 3d). OPEX result is characteristic of capital-intensive preprocessing configurations where the cost of running the plant does not scale proportionally with production volume at low titre.

In the developed scenarios, reducing facility-dependent cost through lower TCI or higher throughput per unit of installed capital is the primary economic lever. Scenario C presents a structurally different cost profile: raw materials account for 75.1% of OPEX (Fig. 3d), dominated by enzymatic hydrolysis reagents and membrane consumables that scale linearly with throughput. Improvements in enzymatic conversion efficiency or enzyme reuse would therefore have directly measurable economic impact in Scenario C, a different engineering priority from Scenarios A and B. Continuous operation reduced OPEX relative to batch across all scenarios by eliminating repeated sterilisation cycles and increasing steady-state throughput per unit of installed capital. For Scenario A the difference is modest ($2.55 M vs. $2.50 M at 5000 L) given the already simple process. For Scenarios B and C, the absolute reduction is larger, though both remain economically non-viable at current experimental titres, as contrasted systematically across scales between the batch economic structures (Table 6) and continuous profiles (Table 7).

#### Operating expenditure structure

Annual OPEX at 5000 L continuous is $2.55 M for Scenario A, $138.8 M for Scenario B, and $101.3 M for Scenario C (Tables 6 and 7; Fig. 3b). In Scenarios A and B, facility-dependent costs such as capital depreciation, maintenance, insurance, and plant overhead, represent the dominant OPEX category (73.2% and 78.1% respectively, as broken down in Fig. 3d). OPEX result is characteristic of capital-intensive preprocessing configurations where the cost of running the plant does not scale proportionally with production volume at low titre. In the developed scenarios, reducing facility-dependent cost through lower TCI or higher throughput per unit of installed capital is the primary economic lever. Scenario C presents a structurally different cost profile: raw materials account for 75.1% of OPEX (Fig. 3d), dominated by enzymatic hydrolysis reagents and membrane consumables that scale linearly with throughput. Improvements in enzymatic conversion efficiency or enzyme reuse would therefore have directly measurable economic impact in Scenario C, a different engineering priority from Scenarios A and B. For Scenarios B and C, the absolute reduction is larger, though both remain economically non-viable at current experimental titres.

**Table 6:** Process simulation SuperPro designer v14 simulation - batch mode comparing all scenario (A, B, C) and scales (1, 100, 1000, 5000 L). All monetary values in USD (2025 prices)

Parameter	Units	Scenario A — Commercial Glucose				Scenario B — Mechanically Pressed Fruit Juice				Scenario C — NF Stream			
Scale	L	1	100	1000	5000	1	100	1000	5000	1	100	1000	5000
CRL1 Production	kg/yr	0.29	3.93	39.27	1.46	0.88	88.30	884.56	4.42	0.78	72.90	460.41	4.12
Total Capital (TCI)	$M	2.09	2.09	3.54	5.90	10.70	10.74	13.07	26.44	25.89	53.89	49.63	169.60
Annual OPEX	$k/yr	1448	1486	2085	2496	3920	3940	4504	7520	6470	11,967	11,202	37,548
Labour Cost	$k/yr	947	974	1215	947	1750	1750	1775	1850	1600	1588	1588	1568
Facility-Dep. Cost	$k/yr	359	359	613	1035	1890	1890	2311	4698	4610	9629	8864	30,277
Raw Materials	$k/yr	~ 0	6	59	294	~ 0	6	56	278	5	435	435	4921
Net Present Value (NPV)	$M	− 11.60	− 11.90	− 16.71	− 19.88	− 32.30	− 32.50	− 36.90	− 61.80	− 54.70	− 101.90	− 95.20	*-316.78*
Return on Invest (ROI).	%	− 60.70	− 62.40	− 50.15	− 32.82	− 27.80	− 27.90	− 25.40	− 19.10	− 16.00	− 13.20	− 13.60	*-13.06*
Unit Production Cost (UPC)	$/kg	4,961,684	378,349	53,080	1711	4,448,554	44,722	5091	1702	8,294,872	164,158	24,341	9102

**Table 7:** Process simulation SuperPro designer v14 outputs: continuous mode comparing all scenario (A, B, C) and scales (1, 100, 1000, 5000 L.

Parameter	Units	Scenario A — Commercial Glucose Chemostat				Scenario B — Mechanically Pressed Fruit Juice Chemostat				Scenario C — NF Stream Chemostat			
Scale	L	1	100	1000	5000	1	100	1000	5000	1	100	1000	5000
CRL1 Production	kg/yr	0.34	40.00	400.51	2151	0.78	78.16	762.08	3,849	5.98	598.48	5984	29,644
Total Capital (TCI)	$M	3.38	5.50	6.00	10.49	21.42	33.64	139.65	608.64	26.16	40.56	56.29	121.99
Annual OPEX	$k/yr	708	1160	1332	2550	6520	9590	33,970	138,750	4880	9100	26,330	101,270
Labour Cost	$k/yr	118	146	170	328	2360	2670	3480	2640	151	223	382	1082
Facility-Dep. Cost	$k/yr	571	982	1070	1867	3800	5970	24,850	108,430	4690	7240	9820	20,570
Raw Materials	$k/yr	~ 0	5	46	248	~ 0	481	4680	24,940	16	1530	15,350	76,060
Net Present Value (NPV)	$M	− 6.21	− 10.01	− 11.27	− 21.04	− 54.34	− 79.95	− 284.62	− 1165	− 42.46	− 76.63	− 202.73	− 750.45
Return on Invest (ROI)	%	− 12.87	− 12.19	− 13.03	− 14.80	− 21.51	− 19.56	− 15.32	− 13.77	− 9.62	− 13.40	− 37.71	− 73.90
Unit Production Cost (UPC)	$/kg	2,082,353	29,000	3326	1186	8,358,974	122,697	44,575	36,048	816,054	15,217	4400	3416

DFC = Direct Fixed Capital. OPEX = Annual Operating Cost. UPC = Unit Production Cost. GM = Gross Margin. PB = Payback Time. IRR = Internal Rate of Return. ~0 denotes a value below $500 yr⁻¹ at the stated precision

For Scenarios B and C, the absolute reduction is larger, though both remain economically non-viable at current experimental titres, as contrasted systematically across scales between the batch economic structures (Table 6) and continuous profiles (Table 7).

#### Unit production cost, break-even titre, and substrate selection

The UPC at 5000 L continuous, obtained from the SuperPro Designer Economic Evaluation Reports (EER), is $1186 kg⁻¹ for Scenario A, $36,049 kg⁻¹ for Scenario B, and $3,416 kg⁻¹ for Scenario C (Table 7). All three values exceed the commodity lipase benchmark of $25 kg⁻¹ by factors of 47, 1,442, and 137 respectively. The UPC of $1186 kg⁻¹ at 5000 L continuous reflects the low secreted CRL1 titre (1.20 g L⁻¹ BCA at steady state, Table 4) rather than a modelling error at this expression level, facility-dependent costs cannot be distributed over sufficient product mass to approach commodity pricing.

The break-even titre (BET) represents the minimum secreted CRL1 concentration at which UPC equals $25 kg⁻¹. BET values were derived directly from each EER OPEX using the chemostat throughput equation defined in Sect. 2.5, using titre as the primary variable. The resulting economic thresholds, operational expenditures, and calculated target titres required for viability across the continuous scenarios are compiled and contrasted in Table 8.

**Table 8:** Break-even titre (BET) calculated from SuperPro Designer EER OPEX values at 5000 L continuous chemostat. D = 0.05 h⁻¹, AOT = 7920 h yr⁻¹, DSP = 0.80, price = $25 kg⁻¹

Scenario	OPEX ($ yr^-1^)	Production (kg yr^-1^)	UPC ($ kg^-1^)	BET (g L⁻¹)
A — Commercial Glucose (continuous)	$2,550,000	2151	$1186	64
B — Mechanically Pressed Fruit Juice (continuous, projected)	$138,756,000	3849	$36,049	3504
C — Enzymatically Hydrolysed NF Sugar Stream (continuous)	$101,275,000	29,644	$3416	2557

D = 0.05 h⁻¹, AOT = 7,920 h yr⁻¹, DSP = 0.80, price = $25 kg⁻¹. Scenario B continuous OPEX is model-based projection from batch stoichiometry; higher uncertainty applies. BET (g L⁻¹) = OPEX ($yr⁻¹) / ($25 kg⁻¹ × 1,584 kg yr⁻¹ per g L⁻¹)

The highest steady-state titres measured under continuous operation were 5.51 g L⁻¹ BCA in Scenario C and 1.20 g L⁻¹ BCA in Scenario A (Table 4). Reaching the corresponding break-even titres would require an approximately 53-fold increase for Scenario A (64 g L⁻¹) and a 464-fold increase for Scenario C (2557 g L⁻¹). Published improvements in CRL1 expression through multicopy cloning and oxygen-limiting conditions in P. pastoris have demonstrated up to ninefold increases in specific productivity (Gasset et al. 2022). Assuming this increase translated proportionally into titre, the Scenario A steady-state titre would reach approximately 10.8 g L⁻¹, still well below its BET of 64 g L⁻¹. This remains far below the break-even titres of Scenarios B and C (3504 and 2557 g L⁻¹), indicating that economic viability would require titre improvements well beyond those currently reported, or a substantial reduction in facility-dependent cost, such as through decoupled preprocessing (Sect. 3.4.2).Waste valorisation is widely promoted as an approach to circular bioeconomy in bioprocesses, with agro-industrial by-products identified as attractive alternative substrates for recombinant protein production (Çaloğlu and Binay 2023). Under EU Directive 2018/851, biowaste classified as a gate-fee material carries zero acquisition cost, which is often presented as an economic advantage in biorefinery configurations. However, the results presented here suggest that feedstock cost is not the binding constraint, even at zero acquisition cost, as in Scenario B, the facility-dependent OPEX dominates, and the BET remains biologically unachievable. This is consistent with findings from comparable recombinant enzyme TEA: in the β-glucosidase system modelled by Ferreira et al. (2018), facility-dependent costs represented 45% of UPC across all scenarios evaluated, with raw material costs playing a secondary role. Waste-derived carbon sources can reduce one cost category but cannot compensate for low secreted protein titres in a facility-dominated cost structure. Çaloğlu and Binay (2023) confirmed that waste-derived substrates can support recombinant laccase enzyme secretion in *P. pastoris under pAOX1*, but no techno-economic analysis was performed in that study, making direct cost comparison impossible.

#### Substrate selection decision

Scenario B presents a higher UPC than Scenario C despite requiring fewer unit operations, with no enzymatic hydrolysis and no nanofiltration step. The main reason for this lies in the nature of the preprocessing: although Scenario B appears simpler, in industrial production it requires processing raw fruit without prior concentration. At 5000 L scale, this means sizing preprocessing equipment for approximately 85.5 million kg of fruit per year, which drives TCI to $608.6 M compared to $122.0 M for Scenario C, where the NF stream arrives already concentrated and at much lower volumes.

A second factor is the difference in data quality between the two scenarios. The Scenario B production figure (3849 kg yr⁻¹) is derived from batch stoichiometry projected to chemostat conditions, with no experimental steady-state validation. In contrast, Scenario C production (29,644 kg yr⁻¹) is based on experimental chemostat data at a measured titre of 5.51 g L⁻¹ BCA. The higher UPC of Scenario B relative to Scenario C may therefore partly reflect the difference in data quality between the two flowsheets, rather than an inherent economic disadvantage of the fruit juice substrate, a distinction that could be resolved through experimental chemostat validation on this feedstock. Gallego-García et al. (2022) identified a similar bottleneck, with low biological conversion yields and high capital requirements limiting economic competitiveness despite the use of a zero-cost FV waste feedstock. The authors evaluated microbial oil production using the oleaginous yeast *Cryptococcus curvatus* and discarded pepper waste from Spanish greenhouses as feedstock, reporting a minimum selling price of €17 kg⁻¹ which is more than tenfold above the commodity vegetable oil benchmark of €1–2 kg⁻¹. Electricity consumed in fermenter agitation and aeration identified as the main contributor to operating costs alongside the cost of supplementary glucose.

Therefore, Scenario B requires further experimental investigation because it involves fewer preprocessing steps and lower reagent costs. The high TCI at 5000 L reflects a structural challenge in modelling integrated biorefinery plants with raw waste feedstocks: the fermenter throughput determines the preprocessing equipment size, and at large scale the volumes of raw fruit required reach industrial processing capacities. Similar to this study´s findings, Aranda-Barradas et al. (2025) describe preprocessing of raw materials as a common cost-determining stage in all SuperPro Designer-based biorefinery TEA studies, and its capital contribution increases disproportionately at large scale when the feedstock arrives unconcentrated. A facility processing both raw FV waste and producing recombinant enzyme would require capabilities spanning two very different industrial sectors, which introduces operational complexity not captured in a single-flowsheet model (Aranda-Barradas et al. 2025). Whether a more concentrated or single-source agrowaste stream could reduce this preprocessing burden to economically feasible levels remains an open question that future experimental and modelling work could address.

### Sensitivity analysis

**Fig. 4:**
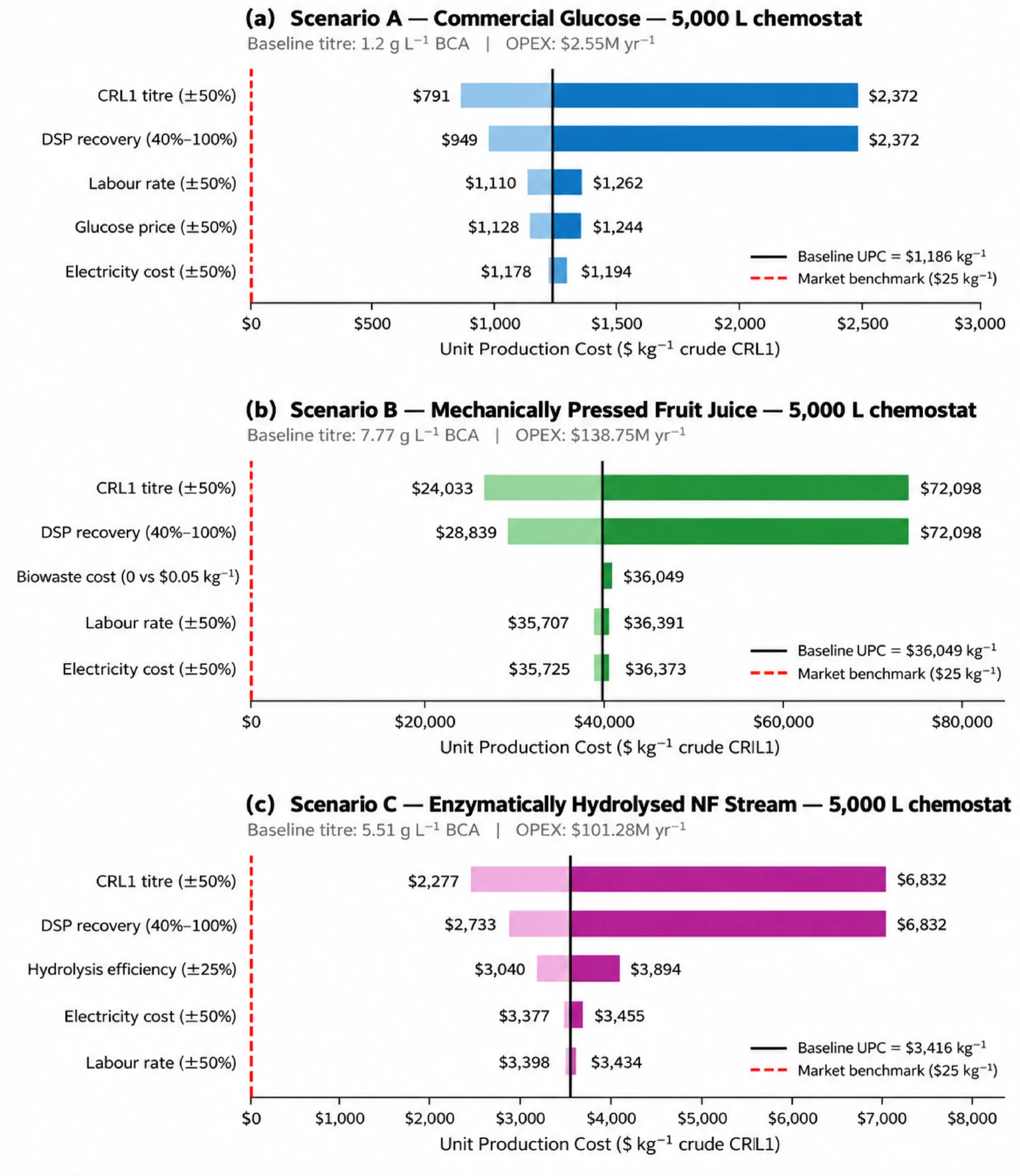
One-at-a-time (OAT) sensitivity analysis at 5000 L continuous chemostat for Scenarios A, B, and C. Parameters varied ± 50% relative to baseline. Bars show change in UPC ($ kg⁻¹) from baseline. Baseline UPC values: Scenario A $1186 kg⁻¹; Scenario B $36,049 kg⁻¹; Scenario C $3416 kg

One-at-a-time (OAT) sensitivity analysis at 5000 L continuous confirmed that secreted CRL1 titre and DSP recovery are the dominant economic levers across all three scenarios (Fig. 4). A 50% reduction in titre doubles the UPC (+ 100%), while a 50% increase reduces it by one third (− 33%), a direct consequence of titre appearing in the denominator of the UPC equation. A ± 50% variation in DSP recovery has an equivalent effect by the same mechanism, since both parameters multiply production in the denominator.

By contrast, a ± 50% variation in electricity cost changes UPC by less than 2% in all scenarios, and a ± 50% variation in labour rate changes UPC by less than 20% in Scenario A and less than 2% in Scenarios B and C. These small sensitivities reflect the low fractions of OPEX attributable to utilities (1.4–2.3%) and labour (1.1–12.9%) at 5,000 L continuous. No combination of reductions in utility cost, labour efficiency, or feedstock acquisition price can close the gap between current UPC and the commodity benchmark of $25 kg. The only economically meaningful levers are improvements in secreted titre and DSP recovery. OAT sensitivity analysis confirmed that secreted protein titre and DSP recovery are the only parameters with meaningful economic leverage. Electricity cost, labour rate, and feedstock acquisition price collectively account for less than 13% of UPC variation in any scenario. This result is consistent with published TEA studies for comparable recombinant enzyme systems (Ferreira et al. 2018; Cunniffe et al. 2025) and extends their findings to waste-derived substrates, where preprocessing capital constitutes an additional fixed cost that amplifies the titre dependency. A structural difference emerges in Scenario C: a ± 25% variation in enzymatic hydrolysis efficiency changes UPC by approximately 11–14%, reflecting the large OPEX fraction attributable to hydrolysis reagents (75.1% of OPEX). This sensitivity is absent in Scenarios A and B, where hydrolysis is not part of the cost structure. Enzyme cost reduction or process intensification of the hydrolysis step would therefore have measurable economic impact specifically in Scenario C.

The feedstock cost structure differed between scenarios in an informative way. In Scenarios A and B, facility-dependent costs (depreciation, maintenance, insurance) dominated OPEX—73% and 78% respectively, confirming that the economic barrier is capital-driven rather than substrate-driven. In Scenario C, raw material costs (principally enzymatic hydrolysis reagents) accounted for 75% of OPEX, identifying enzyme cost reduction as the primary lever for that flowsheet. The zero acquisition cost of Scenario B under the EU biowaste gate-fee model (EU Directive 2018/851) does not offset the preprocessing capital required to convert heterogeneous mixed FV waste at industrial scale. These results are consistent with the TRY framework proposed by Konzock and Nielsen (2024) for microbial fermentation processes. Under low-product-titre conditions, product titre is the main economic driver because it directly affects unit production cost. In contrast, production rate mainly influences capital requirements, while substrate yield has a secondary economic impact unless raw material or processing reagent costs become dominant. This exception is illustrated by Scenario C, where raw material costs account for 75.1% of total OPEX.

### Limitations of the experimental dataset and techno-economic modelling

Residual glucose, fructose and extracellular metabolites were not quantified by HPLC in the experiments presented in this study. This limits the direct assessment of substrate conversion and prevents a detailed kinetic comparison of carbon-source utilisation among the three feedstock scenarios. The experiments were designed primarily to generate comparative process-performance data and input parameters for the techno-economic assessment, rather than to provide a comprehensive kinetic characterisation of each substrate. Initial fermentable-sugar concentrations were therefore established from the compositional analyses available for commercial glucose, mechanically pressed fruit juice and the processed waste-derived sugar stream. These concentrations were used to standardise the fermentation media and parameterise the corresponding process models. Consequently, the experimental comparison focused on biomass formation, extracellular protein production, CRL1 activity and volumetric productivity. Substrate-based yields and conversion values incorporated into the techno-economic models should therefore be interpreted in the context of the assumptions defined for each scenario, rather than as values derived from direct residual-sugar measurements. This constraint is particularly relevant for interpreting the unexpected chemostat performance observed for Scenario C.

The process simulation models presented in the present study show several structural limitations that should be considered when interpreting the TEA outputs, beginning with the fact that all scale-up models are based on single experimental replicates (*n* = 1) at 0.75–7 L. The scale-up approach applied constant specific power input (P/V) and constant superficial gas velocity (vs.), following van’t Riet’s (1979) correlation for oxygen mass transfer in stirred vessels (Doran 2012), as a practical substitute for direct kLa measurement. This was necessary because SuperPro Designer performs steady-state mass and energy balances but does not model gas-liquid hydrodynamics or bubble behaviour directly, so kLa cannot be computed within the software itself. For non-coalescing broth (that is, broth in which dissolved salts and proteins prevent air bubbles from merging readily, typical of fermentation media), this correlation indicates that oxygen transfer depends more strongly on power input than on aeration rate, which is why power input was prioritised whenever both parameters could not be held constant simultaneously across scales. This workaround carries two assumptions that the software itself cannot verify. First, holding gas velocity constant across the roughly 6,700-fold volume increase from 0.75 to 5000 L does not preserve the amount of air delivered per unit broth volume, since tank cross-sectional area and liquid volume scale differently with vessel size; this is an unavoidable geometric consequence of scale-up rather than a modelling choice, and its magnitude can be derived directly from the vessel geometry (Doran 2012). Second, van’t Riet’s correlation assumes geometric similarity between vessels of different size, and mixing time is known to increase with scale even where this similarity is preserved, producing local oxygen and concentration gradients that a process simulator built on steady-state balances cannot represent. Neither assumption was validated experimentally in this study. Direct kLa measurement at an intermediate pilot scale is identified as a priority for future work before the fermentation performance projected at 5000 L can be considered fully validated.

The model does not account for antifoam consumption, which increases with fermenter volume due to greater gas-liquid interfacial area and longer residence time in continuous operation (Ferreira et al. 2018). This represents a minor but systematic underestimation of OPEX at large scale. Additionally, the model assumes a single fixed feedstock composition throughout the annual operating cycle. In practice, FV waste from a municipal market source such as Mercavalencia varies seasonally in sugar concentration, inhibitor profile, and organic nitrogen content. This compositional variability is not captured by the deterministic single-composition simulation and represents a source of uncertainty in the biological performance predictions (Poddar and Scown 2025). This study characterises CRL1 production and secretion but does not evaluate the enzyme’s performance in an applied context, such as hydrolysis of a real substrate, detergent formulation testing, or operational stability under application-relevant conditions. Such testing would be required before the economic conclusions presented here could directly inform a specific end-use application.

The electricity price used in this study ($0.13 kWh⁻¹, Eurostat 2026) represents the medium-term average for industrial consumers in Spain and does not capture the volatility observed in European energy markets following geopolitical disruptions since 2022, when Spanish industrial prices reached €0.25–0.30 kWh⁻¹ in peak periods. At the scales modelled here, electricity represents less than 4% of OPEX across all scenarios, so price volatility has limited impact on the economic conclusions. However, this assumption would not hold for more energy-intensive downstream processing configurations, such as spray drying or high-pressure homogenisation. Biorefinery facilities co-located with waste processing infrastructure could potentially use on-site renewable energy sources, including biogas from anaerobic digestion of the pomace fraction or solar thermal for steam generation, which would reduce both energy cost and carbon footprint but are outside the scope of this deterministic model.

Process water consumption represents a significant utility in Scenarios B and C at large scale. The water tariff applied ($1.85 m⁻³) reflects industrial process water from a central supply, not potable municipal water, and is consistent with published biorefinery TEA assumptions (Humbird et al. 2011). However, Spanish industrial water tariffs vary regionally between €1.50 and €4.50 m⁻³ depending on source and treatment requirements, and large-scale FV waste processing generates significant effluent that would require wastewater treatment, a cost not included in the current model. Outsourcing wastewater and solid waste management to specialist entities, as recommended for early-stage bioproduct facilities (Poddar and Scown 2025), could reduce both capital requirement and operational liability for the preprocessing section.

More broadly, the limitations identified here are consistent with documented challenges in large-scale bioprocess simulation using SuperPro Designer. Aranda-Barradas et al. (2025) highlight that large-scale simulations cannot be fully representative of real operational conditions, as most input data derive from laboratory-scale experiments that lack scalability validation. These authors also identify the integration of multiple sequential unit operations and that integrating multiple sequential unit operations into a single cohesive model is a source of compounding uncertainty (Aranda-Barradas et al. 2025), particularly for processes using heterogeneous waste feedstocks. Equipment cost correlations in SuperPro Designer are derived primarily from chemical process industries and may not accurately reflect the capital cost of novel agro-industrial preprocessing equipment for heterogeneous fruit and vegetable waste streams, for which published cost data are limited. Water and energy consumption at large scale are additionally cited as factors that can determine process feasibility but are often underestimated in simulation-based TEA. The present study is subject to all these limitations, and the economic outputs should be interpreted as order-of-magnitude screening estimates rather than engineering-grade projections.

## Conclusions and future recommendations

This study integrated bench-scale fermentation data with multi-scenario process simulation to evaluate the techno-economic feasibility of recombinant CRL1 production in *P. pastoris* under *pGAP* across three carbon-source scenarios, commercial glucose (Scenario A), mechanically pressed FV juice (Scenario B), and an enzymatically hydrolysed NF sugar stream (Scenario C), at four scales from 1 to 5,000 L, in both batch and continuous chemostat modes. The analysis identifies the dominant cost drivers for each feedstock scenario and provides a quantitative basis for substrate selection within the project MIXMATTERS circular bioeconomy application in Valencia, Spain.

In this comparative feasibility assessment, the effect of continuous chemostat operation depended on the carbon source. On commercial glucose, chemostat increased volumetric productivity 12.6-fold over batch (0.08 vs. 0.006 U L⁻¹ h⁻¹), showing that operating mode strongly affects process economics to a similar extent as feedstock selection. On the NF stream (Scenario C), the productivity advantage of chemostat over batch was not observed, volumetric productivity was lower in chemostat (0.02 U L⁻¹ h⁻¹) than in batch (0.07 U L⁻¹ h⁻¹), suggesting that D = 0.05 h⁻¹ may not represent the productivity optimum for this substrate. This result could also reflect incomplete or preferential sugar utilisation, nutrient imbalance, or the presence of inhibitory compounds generated during enzymatic hydrolysis, which may reduce metabolic activity or biomass-specific CRL1 production (Paes et al. 2021).

At 5000 L continuous, the unit production cost (UPC) was $1186 kg⁻¹ for Scenario A, $36,049 kg⁻¹ for Scenario B, and $3416 kg⁻¹ for Scenario C, all above the commodity lipase benchmark of $25 kg⁻¹. Break-even titres of 64, 3504, and 2557 g L⁻¹ calculated from the verified OPEX values are not achievable with the current *pGAP* strain. OAT sensitivity analysis confirmed that titre and DSP recovery are the only parameters with substantial effect on unit production cost across all three scenarios; electricity, labour, and feedstock acquisition price collectively account for less than 13% of UPC variation in any scenario (up to 12.5% in Scenario A). Facility-dependent costs dominated OPEX in Scenarios A and B (73% and 78% respectively), while raw material costs dominated in Scenario C (75%), driven by enzymatic hydrolysis reagents. Zero feedstock acquisition cost under the EU biowaste gate-fee model did not offset preprocessing capital in Scenario B. The Scenario B continuous model carries the highest uncertainty of the three flowsheets, as it is based on batch stoichiometry with no chemostat validation. Its modelled UPC therefore cannot be compared reliably against Scenario C. What the model does establish is that mechanical pressing is the lower preprocessing intensity route of the two waste scenarios and that the high TCI of Scenario B at 5000 L is a consequence of processing raw unconcentrated fruit at industrial throughput, not of the substrate itself. Experimental chemostat validation on a compositionally consistent biowaste source could be considered as a next step for this scenario.

This work provides a quantitative framework for guiding substrate and process mode selection at the early stages of waste-to-enzyme bioprocess development. To the best of our knowledge, techno-economic datasets spanning this many combinations of feedstock, operating mode, and scale, twenty-four process configurations in total, are uncommon in the published literature on waste-derived recombinant protein production, where TEA studies more typically report a single scenario or scale. Accordingly, the models, EER files, and sensitivity analysis scripts developed here are provided in full as supplementary material, rather than only the aggregated results, to enable direct adaptation of this framework to other recombinant enzyme systems or agrowaste feedstocks without requiring a comparable model to be rebuilt from first principles.

At current expression levels, preprocessing capital and secreted titre are the main economic constraints, not feedstock cost. Early-stage TEA frameworks of this type, grounded in experimental bench-scale data, can support feedstock selection and process mode decisions before pilot-scale investment is made, contributing to the development of economically grounded circular bioeconomy strategies for waste-to-enzyme applications. However, replicated cultivation data, direct residual-sugar measurements, and pilot-scale validation will be required to reduce uncertainty before the models can support detailed industrial design or investment decisions.

## Supplementary Information

Below is the link to the electronic supplementary material.

Supplementary Material 1

Supplementary Material 2

Supplementary Material 3

## Data Availability

The datasets supporting the conclusions of this article are included within the article and its additional files. The SuperPro Designer v14 process models, Economic Evaluation Reports (PDF), and sensitivity analysis scripts supporting this study are deposited in a Zenodo repository (https://doi.org/10.5281/zenodo.21620393).

## Abbreviations

AOT: Annual operating time
BCA: Bicinchoninic acid
BET: Break-even titre
CAPEX: Capital expenditure
CIP: Clean-in-place
CRL1: Candida rugosa lipase type 1
D: Dilution rate (h⁻¹)
DCW: Dry cell weight
DO: Dissolved oxygen
DSP: Downstream processing
EER: Economic evaluation report
EU: European Union
FV: Fruit and vegetable
pGAP: Glyceraldehyde-3-phosphate dehydrogenase promoter
k_La_: Volumetric oxygen mass transfer coefficient
NF: Nanofiltration / nanofiltered
NPV: Net present value
OAT: One-at-a-time
OD: Optical density
OPEX: Annual operating expenditure
PAN: Primary amino nitrogen
P/V: Specific power input (W m⁻³)
ROI: Return on investment
SDS-PAGE: Sodium dodecyl sulphate–polyacrylamide gel electrophoresis
TCI: Total capital investment
TEA: Techno-economic analysis
TFF: Tangential-flow filtration
TRY: Titer, rate, and yield
UPC: Unit production cost
vs: Superficial gas velocity (m s⁻¹)
vvm: Volume of gas per volume of liquid per minute
WACC: Weighted average cost of capital
Y_x/s_: Biomass yield on substrate (g g⁻¹)
Yp/s: Product yield on substrate (g g⁻¹)

## Declarations

### Use of artificial intelligence tools

The authors acknowledge the use of Claude to assist with English language editing, grammar, and stylistic consistency. Its use was limited to language refinement and did not involve the generation of scientific content, data analysis, results, or interpretations. All scientific decisions, analyses, interpretations, and conclusions remain entirely the responsibility of the authors.
