# Supplementary material for: What drives the cost of biowaste-to-lipase production? Linking titre, rate and yield to techno-economic performance in batch-to-continuous Pichia pastoris fermentation
Source: Bioresour Bioprocess. 2026 Oct 29;13(1):160. doi: 10.1186/s40643-026-01153-x (PMC13719962; doi:10.1186/s40643-026-01153-x)
Supplement: Supplementary file 1 [file 40643_2026_1153_MOESM1_ESM.docx]

**What drives the cost of biowaste-to-lipase production? Linking titre, rate and yield to techno-economic performance in batch-to-continuous *Pichia pastoris* fermentation**

Ana G. Del Hierro ^1,2,3^, Violine Raynal ^1^, Ruth Ordóñez^1,^ Fuensanta Verdú-Navarro ^3^ & Eoin Casey ^1,2 *^

1. School of Chemical and Bioprocess Engineering, University College Dublin, Belfield, Dublin 4, Ireland
2. BiOrbic, Research Ireland Centre for Bioeconomy, Dublin, Ireland
3. Bioprocessing R&D Department, Bionet, Parque Tecnológico Fuente Álamo, Fuente Álamo, Spain

*Corresponding Author: Eoin Casey, [eoin.casey@ucd.ie](mailto:eoin.casey@ucd.ie)

## 1. **Supplementary Material**

**The following supplementary files are available online alongside this article.**

**Supplementary Table S1.** Break-even titre (BET) calculations at 5,000 L continuous chemostat derived from verified EER OPEX values, including the production factor derivation.

**Supplementary Table S2.** One-at-a-time (OAT) sensitivity analysis: full numerical values for all parameters and scenarios at 5,000 L continuous chemostat.

**Supplementary Table S3.** Biomass yield in the Scenario C chemostat: experimental value and model coefficient

**Supplementary EER (Files S1–S24).** SuperPro Designer v14 Economic Evaluation Reports (PDF) for all 24 process configurations evaluated in this study (3 scenarios × 2 operating modes × 4 scales). Deposited in Zenodo repository.

**Supplementary Script S1.** Python v3.10 script (tea_figures.py) for generating all sensitivity analysis figures and UPC calculations reported in this study.

**Figure S1.** SuperPro Designer v14 process flowsheet at 5,000 L — Scenario A (commercial glucose), continuous mode. The flowsheet illustrates the four process sections: media preparation, inoculum preparation, continuous fermentation (batch phase FR-101 followed by continuous chemostat FR-102), and downstream processing (centrifugation DS-101 and microfiltration MF-101).

**Figure S2.** SuperPro Designer v14 process flowsheet at 5,000 L — Scenario B (mechanically pressed fruit juice), continuous mode. The preprocessing section (solids storage SL-101, shredding SR-101, grinding GR-101, leaching SMSX-101) precedes the fermentation and downstream sections shown in Figure S1.

**Figure S3.** SuperPro Designer v14 process flowsheet at 5,000 L — Scenario C (enzymatically hydrolysed NF sugar stream), continuous mode. The preprocessing section includes enzymatic hydrolysis (BR-101) and nanofiltration (UF-101) upstream of fermentation
